# Phototropin Interactions with SUMO Proteins

**DOI:** 10.1093/pcp/pcab027

**Published:** 2021-02-17

**Authors:** Justyna Łabuz, Olga Sztatelman, Dominika Jagiełło-Flasińska, Paweł Hermanowicz, Aneta Bażant, Agnieszka Katarzyna Banaś, Filip Bartnicki, Aleksandra Giza, Anna Kozłowska, Hanna Lasok, Ewa Sitkiewicz, Weronika Krzeszowiec, Halina Gabryś, Wojciech Strzałka

**Affiliations:** Laboratory of Photobiology, Malopolska Centre of Biotechnology, Jagiellonian University, Gronostajowa 7A, Kraków 30-387, Poland; Department of Plant Biotechnology, Faculty of Biochemistry, Biophysics and Biotechnology, Jagiellonian University, Gronostajowa 7, Kraków 30-387, Poland; Institute of Biochemistry and Biophysics, Polish Academy of Sciences, Pawińskiego 5a, Warszawa 02-106, Poland; Department of Plant Biotechnology, Faculty of Biochemistry, Biophysics and Biotechnology, Jagiellonian University, Gronostajowa 7, Kraków 30-387, Poland; Laboratory of Photobiology, Malopolska Centre of Biotechnology, Jagiellonian University, Gronostajowa 7A, Kraków 30-387, Poland; Department of Plant Biotechnology, Faculty of Biochemistry, Biophysics and Biotechnology, Jagiellonian University, Gronostajowa 7, Kraków 30-387, Poland; Department of Plant Biotechnology, Faculty of Biochemistry, Biophysics and Biotechnology, Jagiellonian University, Gronostajowa 7, Kraków 30-387, Poland; Department of Plant Biotechnology, Faculty of Biochemistry, Biophysics and Biotechnology, Jagiellonian University, Gronostajowa 7, Kraków 30-387, Poland; Department of Plant Biotechnology, Faculty of Biochemistry, Biophysics and Biotechnology, Jagiellonian University, Gronostajowa 7, Kraków 30-387, Poland; Laboratory of Photobiology, Malopolska Centre of Biotechnology, Jagiellonian University, Gronostajowa 7A, Kraków 30-387, Poland; Laboratory of Photobiology, Malopolska Centre of Biotechnology, Jagiellonian University, Gronostajowa 7A, Kraków 30-387, Poland; Institute of Biochemistry and Biophysics, Polish Academy of Sciences, Pawińskiego 5a, Warszawa 02-106, Poland; Department of Plant Biotechnology, Faculty of Biochemistry, Biophysics and Biotechnology, Jagiellonian University, Gronostajowa 7, Kraków 30-387, Poland; Department of Plant Biotechnology, Faculty of Biochemistry, Biophysics and Biotechnology, Jagiellonian University, Gronostajowa 7, Kraków 30-387, Poland; Department of Plant Biotechnology, Faculty of Biochemistry, Biophysics and Biotechnology, Jagiellonian University, Gronostajowa 7, Kraków 30-387, Poland

**Keywords:** Arabidopsis, Blue light, Chloroplast movements, Phototropin, Phototropism, Sumoylation

## Abstract

The disruption of the sumoylation pathway affects processes controlled by the two phototropins (phots) of *Arabidopsis thaliana*, phot1 and phot2. Phots, plant UVA/blue light photoreceptors, regulate growth responses and fast movements aimed at optimizing photosynthesis, such as phototropism, chloroplast relocations and stomatal opening. Sumoylation is a posttranslational modification, consisting of the addition of a SUMO (SMALL UBIQUITIN-RELATED MODIFIER) protein to a lysine residue in the target protein. In addition to affecting the stability of proteins, it regulates their activity, interactions and subcellular localization. We examined physiological responses controlled by phots, phototropism and chloroplast movements, in sumoylation pathway mutants. Chloroplast accumulation in response to both continuous and pulse light was enhanced in the E3 ligase *siz1* mutant, in a manner dependent on phot2. A significant decrease in phot2 protein abundance was observed in this mutant after blue light treatment both in seedlings and mature leaves. Using plant transient expression and yeast two-hybrid assays, we found that phots interacted with SUMO proteins mainly through their N-terminal parts, which contain the photosensory LOV domains. The covalent modification in phots by SUMO was verified using an Arabidopsis sumoylation system reconstituted *in bacteria* followed by the mass spectrometry analysis. Lys 297 was identified as the main target of SUMO3 in the phot2 molecule. Finally, sumoylation of phot2 was detected in Arabidopsis mature leaves upon light or heat stress treatment.

## Introduction

Phototropins (phots) are plant blue/UVA photoreceptors, which control growth responses and fast movements, such as stomatal opening and chloroplast relocations (Christie 2007, Banaś et al. 2012). Two phot genes are found in the genome of *Arabidopsis thaliana*: *PHOT1* (Liscum and Briggs 1995) and *PHOT2* (Jarillo et al. 2001, Kagawa 2001). They share highly redundant functions; however, phot1 is more sensitive to light than phot2 (Sakai et al. 2001, Harada and Shimazaki 2007, Hart et al. 2019). Both phots are responsible for phototropic bending (Sakai et al. 2001) in seedlings. In mature leaves, they both control stomatal opening (Kinoshita et al. 2001) and the chloroplast accumulation response (Sakai et al. 2001). Full chloroplast avoidance (Jarillo et al. 2001, Kagawa 2001, Sakai et al. 2001) and chloroplast dark positioning (Suetsugu et al. 2005) are triggered only by phot2. Gene expression is regulated by phots to a very small extent (Chen et al. 2008, Lehmann et al. 2011).

The phot molecule consists of two parts. The photosensory N-terminal part comprises LOV1 and LOV2 (light, oxygen and voltage regulated) domains, which carry flavin mononucleotide chromophores. The C-terminal part contains a light activated Ser/Thr kinase (Christie 1998). In darkness, the LOV2 domain acts as an inhibitor of the constitutive kinase activity (Matsuoka and Tokutomi 2005). Light perceived by the LOV domains triggers conformational changes, leading to kinase activation (Crosson and Moffat 2001) and receptor autophosphorylation, which is important for signal transduction (Christie 1998, Sakai et al. 2001). Only a few phot kinase substrates are known (Christie et al. 2011, Demarsy et al. 2012, Takemiya et al. 2016).

Phots localize mainly to the plasma membrane (Sakamoto and Briggs 2002, Kong et al. 2006); however, they can be detected also at the chloroplast outer envelope (Kong et al. 2013). After blue light irradiation, a fraction of phot1 moves to the cytosol (Sakamoto and Briggs 2002), while phot2 associates with the Golgi apparatus (Kong et al. 2006, Aggarwal et al. 2014). Internalization of phot1 from the plasma membrane may result from photoreceptor mono/multiubiquitination by a CULLIN3-based E3 ubiquitin ligase, CRL3^NPH3^. Polyubiquitination of phot1 by CRL3^NPH3^ promotes its degradation in the 26S proteasome (Roberts et al. 2011). Phot2 has been reported to interact with COP1 (CONSTITUTIVE PHOTOMORPHOGENIC 1) (Jeong et al. 2010), an E3 ubiquitin ligase involved in the control of stomatal aperture in a manner epistatic to phots (Mao et al. 2005). Thus, posttranslational modifications, such as ubiquitination and phosphorylation, are important for phot functioning.

Sumoylation is a highly dynamic and reversible posttranslational modification, which consists in attachment of a small protein (about 10 kDa) called SUMO (SMALL UBIQUITIN-RELATED MODIFIER) to a target protein. Expressed sequence tags of four SUMO genes were found in Arabidopsis: *SUM1*, *SUM2*, *SUM3* and *SUM5*. SUMO1 and SUMO2 localize to the nucleus and the cytoplasm, where they can recognize and modify membrane substrates (Kurepa et al. 2003). The double *sum1sum2* mutant is embryonic lethal (Saracco et al. 2007). *SUM1* and *SUM2* share high sequence similarity. Their transcripts are abundant in most Arabidopsis tissues and cells, including light- and dark-grown seedlings (Kurepa et al. 2003). The transcript of *SUM3* is poorly expressed. It is found at higher levels in roots (Saracco et al. 2007). *SUM5* mRNA is detected mostly in generative tissues. Mature SUMO proteins bear a diglycine (GG) motif at the C-terminus. SUMO terminal carboxyl group is conjugated to the ε-amine lysine group of the target protein. The sumoylation consensus motif consists of ΨKXE/D, where Ψ is a large hydrophobic residue: X, any amino acid; E/D, glutamate or aspartate (Elrouby and Coupland 2010). SUMO is attached in a multi-step process similar to ubiquitination *(*for a review see Verma et al. 2018). In Arabidopsis, SUMO activation is performed by the E1 enzyme consisting of a large subunit SAE2 (SUMO E1 ACTIVATING ENZYME2) and a small subunit SAE1a or SAE1b. Next, SUMO is transferred to the E2 SUMO-conjugating enzyme, encoded by one functional gene *SCE1a* (Saracco et al. 2007). The *sce1a* mutant and the mutant in the SAE2 subunit are embryonic lethal (Saracco et al. 2007). Finally, SUMO is transferred from SCE1 to the target lysine residue (Kurepa et al. 2003), which is facilitated by the activity of SUMO E3 ligases and modulated by SUMO E4 ligases. In Arabidopsis, two E3 ligases, SIZ1 (SAP and Miz1) (Miura et al. 2005) and MMS21 [METHYL METHANE SULFONATE SENSITIVITY 21 (Huang et al. 2009), named also HPY2 (HIGHPLOIDY2) (Ishida et al. 2009)], are not essential for SUMO conjugation to substrates. However, their absence causes dwarfism. The double *siz1mms21* mutant is embryonic lethal (Ishida et al. 2012). SUMO E4 ligases, PIAL1 and PIAL2 [PROTEIN INHIBITOR OF ACTIVATED STAT (PIAS) LIKE1 and 2], promote the formation of poly-SUMO chains (Tomanov et al. 2014). Apart from covalent conjugation, SUMO may interact non-covalently with target proteins by SUMO interaction motifs (SIMs), which play a regulatory role (Verma et al. 2018).

Sumoylation alters protein subcellular localization, function, activity and interactions with other protein partners (Augustine and Vierstra 2018). It modulates plant responses to abiotic and biotic stresses, such as heat, drought, osmotic stress and pathogen attack *(*for a review see Elrouby 2015, Benlloch and Lois 2018). Sumoylation was shown to participate also in light signaling. The SIZ1 ligase negatively regulates photomorphogenesis by mediating SUMO modification in COP1, which increases its E3 ubiquitin ligase activity. Light decreases the level of COP1 sumoylation and reduces COP1 activity, resulting in the transcription activation (Lin et al. 2016). Sumoylation of phytochrome B, a red/far-red photoreceptor, is enhanced by red light, leading to its inactivation and a decrease of red light-induced photomorphogenic responses: hypocotyl elongation and cotyledon opening (Sadanandom et al. 2015).

In this work, we examined the interactions of phots with SUMO proteins and investigated if disruption of the sumoylation pathway influenced responses controlled by these photoreceptors.

## Results

Chloroplast movements and phototropism were examined in Arabidopsis SUMO mutants: *sum1*, *sum2*, *sum3* and *sum5* and E3 ligase mutants: *siz1* and *mms21*. Phot functions are partly redundant; thus, to differentiate phot1- from phot2-dependent pathways, double mutant plants were prepared: *phot1siz1, phot1mms21*, *phot2siz1* and *phot2mms21*.

### Chloroplast movements in leaves of sumoylation pathway mutants

**Fig. 1:**
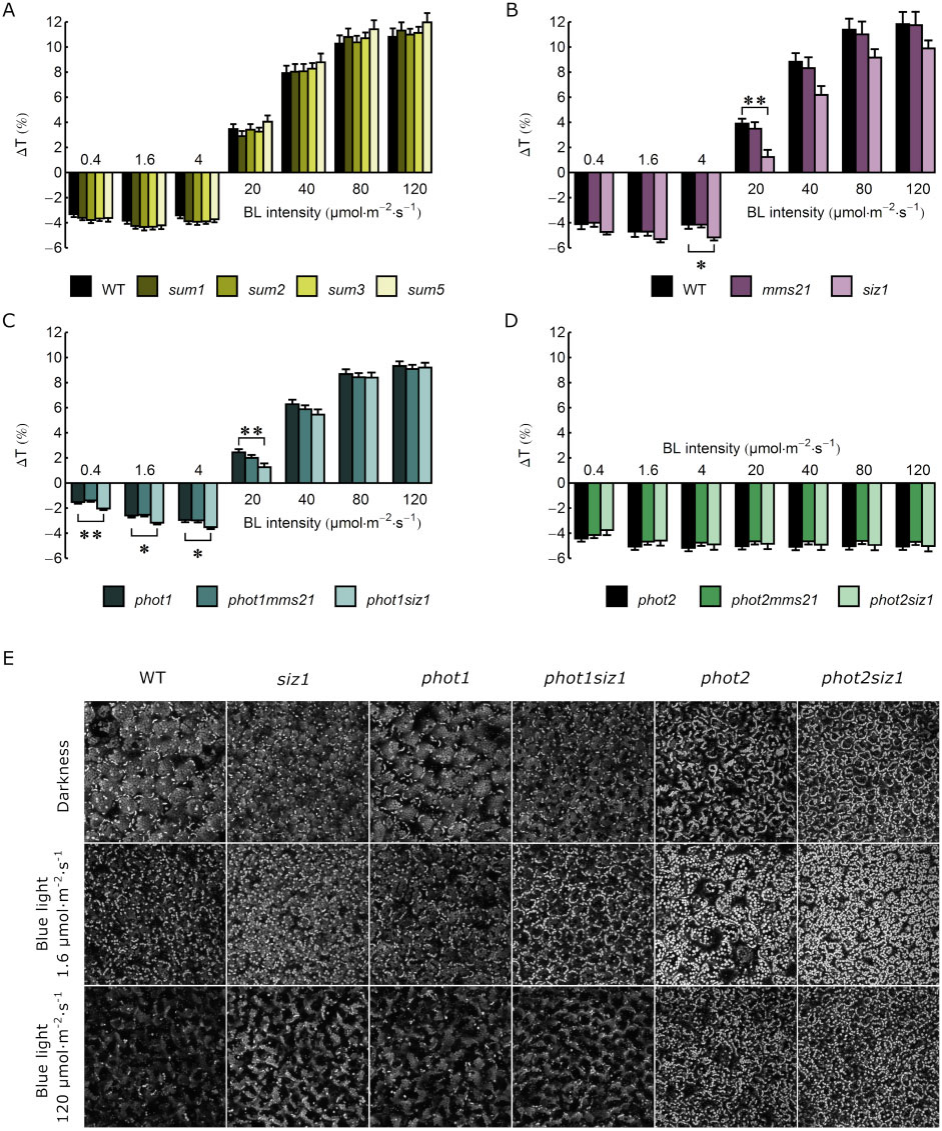
Amplitudes of transmittance changes ΔT due to chloroplast movements in leaves illuminated with continuous blue light of increasing intensity (0.4–120 µmol m^−2^ s^−1^) in (A) *sum* mutants, (B) *siz1* and *mms21* ligase mutants, (C) *phot1siz1* and *phot1mms21* mutants and (D) *phot2siz1* and *phot2mms21* mutants. Asterisks indicate statistically significant differences between mutant lines and the control (*phot1* for *phot1mms21* and *phot1siz1*, *phot2* for *phot2mms21* and *phot2siz1*, the wild type for other lines), as tested with the Dunnett’s test (*P = 0.01–0.05; **P = 0.001–0.01). Error bars = SE. (E) Blue light-induced chloroplast arrangements in palisade cells of Arabidopsis leaves of wild-type, *siz1*, *phot1, phot2*, *phot1siz1* and *phot2siz1* plants. Leaves were kept in darkness or irradiated with blue light (LED 460 nm) of 1.6 or 120 µmol m^−2^ s^−1^ for 50 min. Chloroplast arrangements were then examined under a confocal microscope, using chlorophyll autofluorescence (633 nm excitation, 661–721 nm emission). Maximum intensity projections were calculated from Z-stacks, which spanned whole depth of the epidermis and palisade parenchyma, starting from the leaf upper surface. The fluorescence from chloroplasts located at the bottom of palisade cells is less intense due to attenuation of excitation light.

**Fig. 2:**
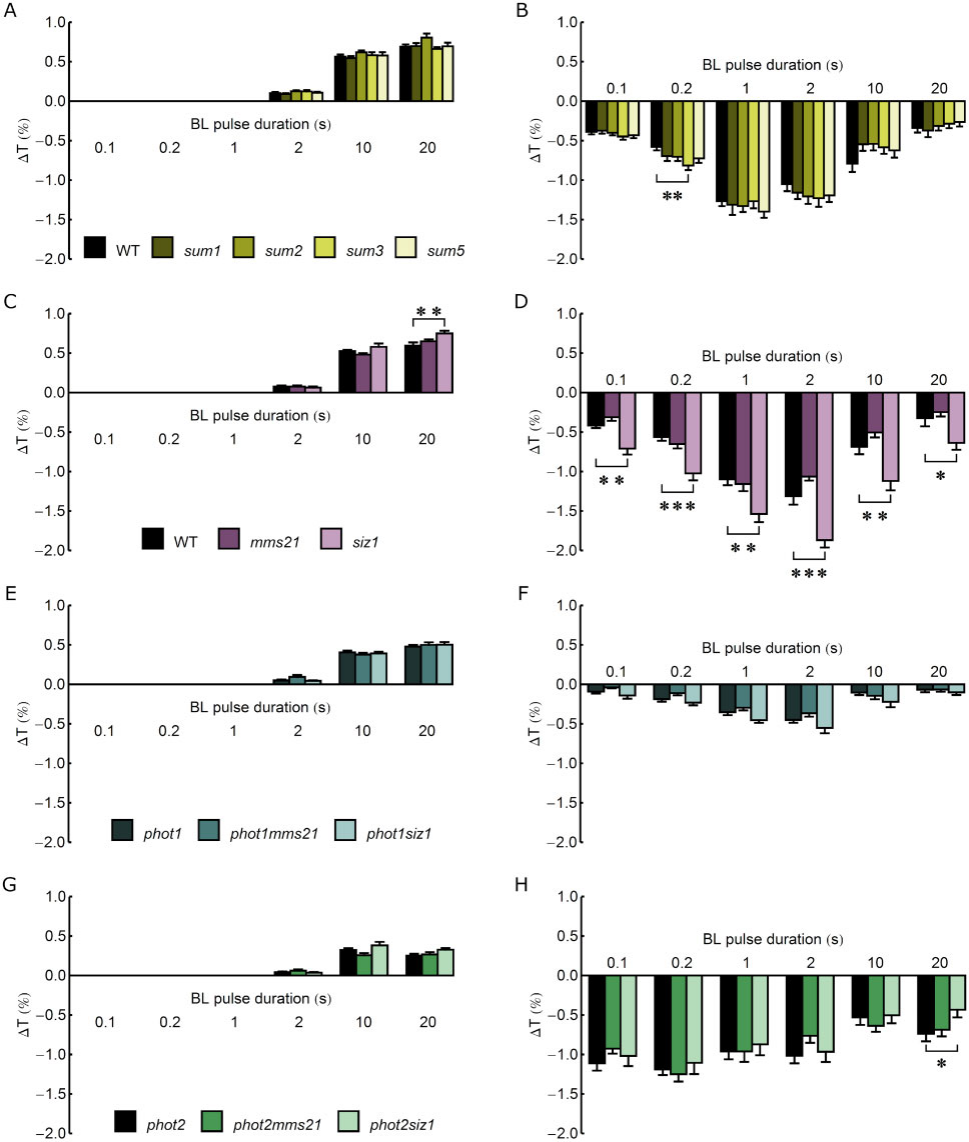
Amplitudes of transmittance changes ΔT due to chloroplast movements triggered by 0.1–20 s long pulses of 120 µmol m^−2^ s^−1^ blue light in (A and B) *sum* mutants, (C and D) *siz1* and *mms21* ligase mutants, (E and F) *phot1siz1* and *phot1mms21* mutants and (G and H) *phot2siz1* and *phot2mms21* mutants. The values were calculated for the transient avoidance (A, C, E and G) and accumulation responses (B, D, F and H). Asterisks indicate statistically significant differences between mutant lines and the control (*phot1* for *phot1mms21* and *phot1siz1*, *phot2* for *phot2mms21* and *phot2siz1*, the wild type for other lines), as tested with the Dunnett’s test (*P = 0.01–0.05; **P = 0.001–0.01, ***P < 0.001). Error bars = SE.

The morphology of 4-week-old wild-type, single and double mutant Arabidopsis plants is shown in Supplementary Fig. S1. In our growth conditions of a short day and relatively low light intensity, *mms21, phot1mms21* and *phot2mms21* did not substantially differ in the size of rosette leaves from wild-type plants, while *siz1, phot1siz1* and *phot2siz1* were smaller. Chloroplast responses to blue light were investigated using the photometric method. Changes in rosette leaf transmittance, indicative of chloroplast relocations (a decrease corresponds to chloroplast accumulation, an increase to chloroplast avoidance), were assessed after the irradiation of dark-adapted leaves with continuous blue light of increasing intensities of 0.4, 1.6, 4, 20, 40, 80, and 120 µmol m^−2^ s^−1^ (Fig. 1, averaged curves in Supplementary Fig. S2). A shift toward chloroplast accumulation was observed in the *siz1* mutant, as the amplitude of accumulation was greater (significantly at 4 µmol m^−2^ s^−1^) and that of avoidance was smaller (significantly at 20 µmol m^−2^ s^−1^) than in the wild type (Fig. 1B). Similarly, the *phot1siz1* mutant showed significantly greater amplitudes of chloroplast accumulation at light intensities of 0.4–4 µmol m^−2^ s^−1^ and smaller avoidance at 20 µmol m^−2^ s^−1^ than *phot1*. No differences were observed between the *phot2* and *phot2siz1* mutants (Fig. 1D). To further analyze chloroplast arrangements in mutants with the *siz1* background, the palisade parenchyma of wild-type, *siz1*, *phot1*, *phot2*, *phot1siz1* and *phot2siz1* dark-adapted plants was examined using a confocal microscope (Fig. 1E). In spite of differences in the leaf size (Supplementary Fig. S1), chloroplast dark positioning was similar in plants bearing the *siz1* mutation and the respective control lines. In leaves of wild type and *siz1*, as well as *phot1* and *phot1siz1*, chloroplasts gathered at the bottom of palisade cells, while in dark-adapted *phot2* and *phot2siz1* leaves, chloroplasts also gathered near the upper periclinal cell wall (Fig. 1E). Transmittance changes induced by 0.1–20 s long pulses of blue light (120 µmol m^−2^ s^−1^) are shown in Fig. 2, the rates in Supplementary Fig. S3 and averaged curves in Supplementary Figs. S4–S7. As for continuous light, the effects of the *siz1* mutation were observed. The amplitudes of transient chloroplast avoidance and accumulation (Fig. 2C, D), as well as the velocities of movements (Supplementary Fig. S3C, D), were significantly higher in *siz1* than in the wild type. The *phot1siz1* mutant showed a slightly stronger accumulation amplitude when compared to *phot1* (Fig. 2F). By contrast, a smaller amplitude of chloroplast accumulation was observed in the *phot2siz1* mutant than in *phot2* plants (significantly at 20 s, Fig. 2H).

To check whether photoreceptor abundance was altered in sumoylation pathway mutants, phot levels were investigated in leaves, which were dark-adapted overnight or treated additionally with blue light of 120 µmol m^−2^ s^−1^ for 3 h (Supplementary Fig. S8A, B). In darkness, PHOT1 levels did not differ between the wild-type and sumoylation mutants. Blue light downregulated PHOT1 expression in all lines tested, and this effect was significantly larger for the *siz1* mutant (Supplementary Fig. S8A). In darkness, the PHOT2 level was similar in most lines, with the exception of *sum5*. After blue light treatment, PHOT2 expression in mutants was also comparable to that in the wild type, except for *siz1* (Supplementary Fig. S8B) where it was significantly lower. Phot mRNA levels were investigated to examine how they correlate with the observed changes in protein expression. Blue light downregulated *PHOT1* (Supplementary Fig. S8C) and upregulated *PHOT2* (Supplementary Fig. S8D) in all analyzed lines.

### Phototropic bending in seedlings of sumoylation pathway mutants

**Fig. 3:**
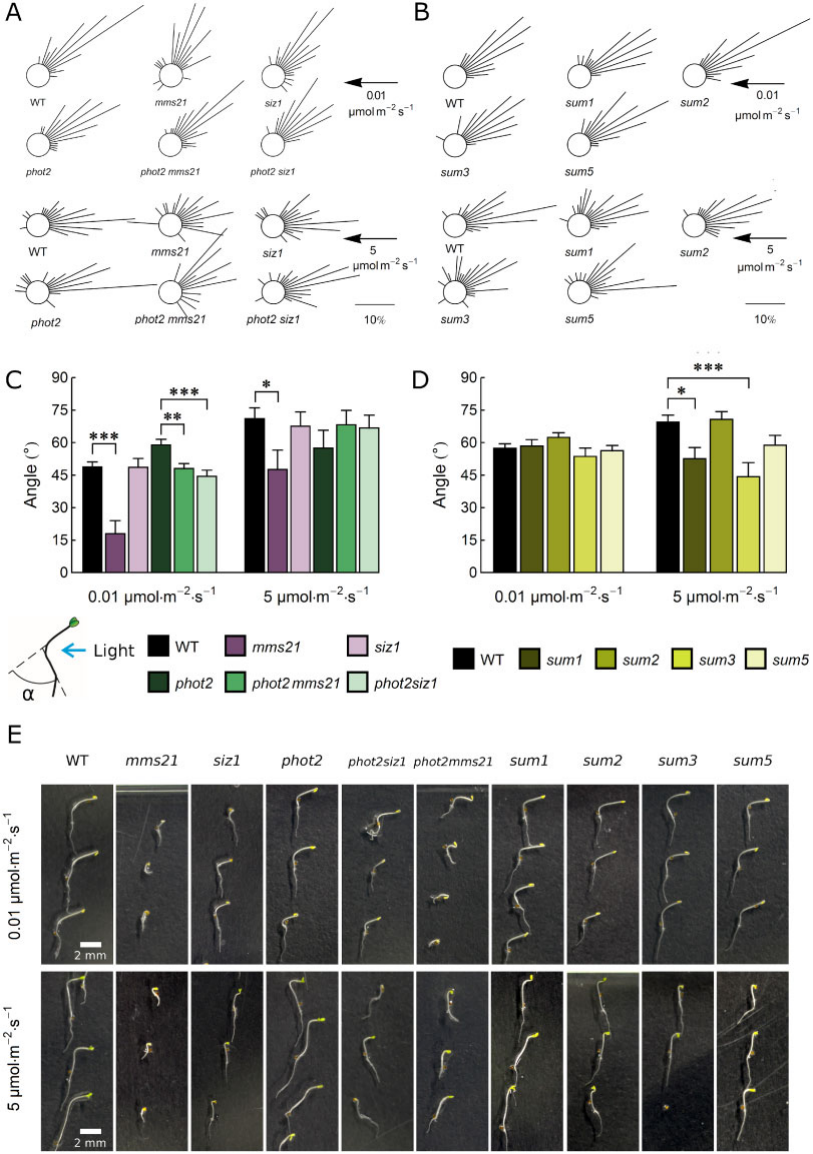
Phototropic bending measured on 3-day-old etiolated seedlings after 12-h-long treatment with blue light of 0.01 or 5 µmol m^−2^ s^−1^ in the wild type and *sum*, *siz1*, *mms21*, *phot2siz1*, *phot2mms21* mutants. (A and B) Circular histograms (Zeidler 2016) of seedlings representing phototropic bending angles sorted into classes (e.g. 0–10°, 11–20°), normalized to the total number of seedlings from each line. The length of the line represents the percentage of seedlings in a specific angle class. The scale bar represents 10% of the seedlings. The arrow indicates the light direction and intensity. (C and D) Averaged phototropic angles. Asterisks indicate significant differences between mutant and control lines (*phot2* for *phot2mms21* and *phot2siz1*, the wild type for other lines), as tested with the Dunnett’s test (*P = 0.01–0.05; **P = 0.001–0.01, ***P < 0.001). Sixty seedlings were measured in each group. Error bars = SE. The seedling drawing shows how the bending angle is defined. (C) Examples of 3-day-old seedlings used for calculation of phototropic bending after 12-h-long treatment with blue light.

Phototropic bending was investigated in 3-day-old etiolated seedlings after treatment with 0.01 or 5 µmol m^−2^ s^−1^ of blue light for 12 h (Fig. 3). These light intensities activate only phot1, which is highly accumulated in etiolated seedlings (Sakai et al. 2001). Bending was reduced in *mms21* at both light intensities as compared to the wild type. At 0.01 µmol m^−2^ s^−1^, the angles were smaller in *phot2mms21* and *phot2siz1* than in the *phot2* control line (Fig. 3A, C, E). However, the growth of *mms21* seedlings was retarded. The phototropic bending was reduced in *sum1* and *sum3* at 5 µmol m^−2^ s^−1^ as compared to the wild type (Fig. 3B, D, E).

To check if the differences in phototropic bending are connected to changes in phot expression resulting, e.g. from altered protein stability, their levels were determined in the investigated mutants (Supplementary Fig. S9A, B). As compared to darkness, the levels of PHOT1 did not change significantly after blue light treatment of 0.01 µmol m^−2^ s^−1^ but decreased four times after irradiation with 5 µmol m^−2^ s^−1^ of blue light (Supplementary Fig. S9A). No statistically significant differences in PHOT1 levels were observed between wild-type plants and single mutants of the sumoylation pathway, irrespective of the light treatment (Supplementary Fig. S9A). In darkness, PHOT2 expression was comparable in all investigated lines except for the *siz1* mutant, which showed significantly lower PHOT2 levels (Supplementary Fig. S9B). After light treatments, the PHOT2 level was lower in the *siz1* mutant than in other lines tested (Supplementary Fig. S9B). At the mRNA level, blue light of 5 µmol m^−2^ s^−1^ downregulated *PHOT1* in all tested lines (Supplementary Fig. S9C), whereas *PHOT2* levels were enhanced in the wild-type and *sum* mutants (Supplementary Fig. S9D).

### Phots interact with components of the sumoylation pathway

**Fig. 4:**
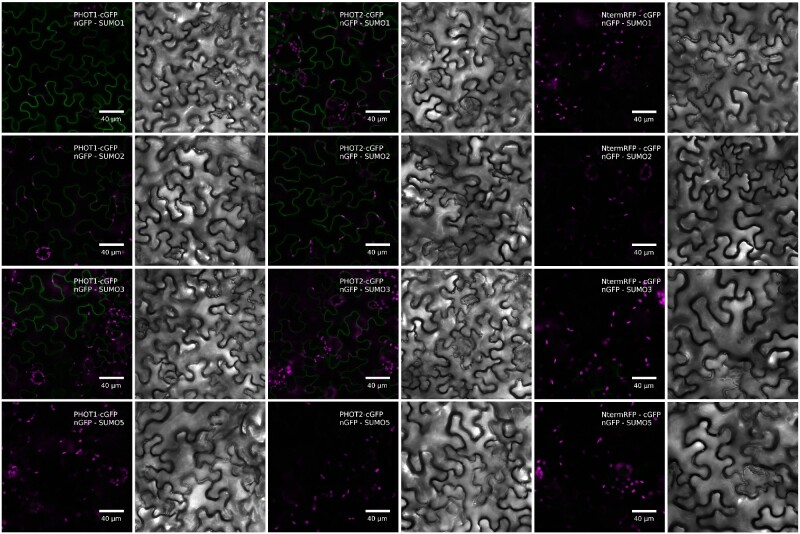
Confocal microscopy images of *N. benthamiana* epidermal cells transiently co-expressing phots fused with the cGFP fragment and SUMO isoforms fused with the N-terminal fragment of GFP (nGFP). Control plants expressed the first 150 amino acids from the N-terminal part of the red fluorescent protein fused with the C-terminal GFP fragment and the N-terminal GFP fragment fused with SUMO isoforms. Chlorophyll autofluorescence is in magenta and reconstituted GFP fluorescence is in green. Gray scale images show transmitted light. The results represent one of the three independent biological replicates.

**Fig. 5:**
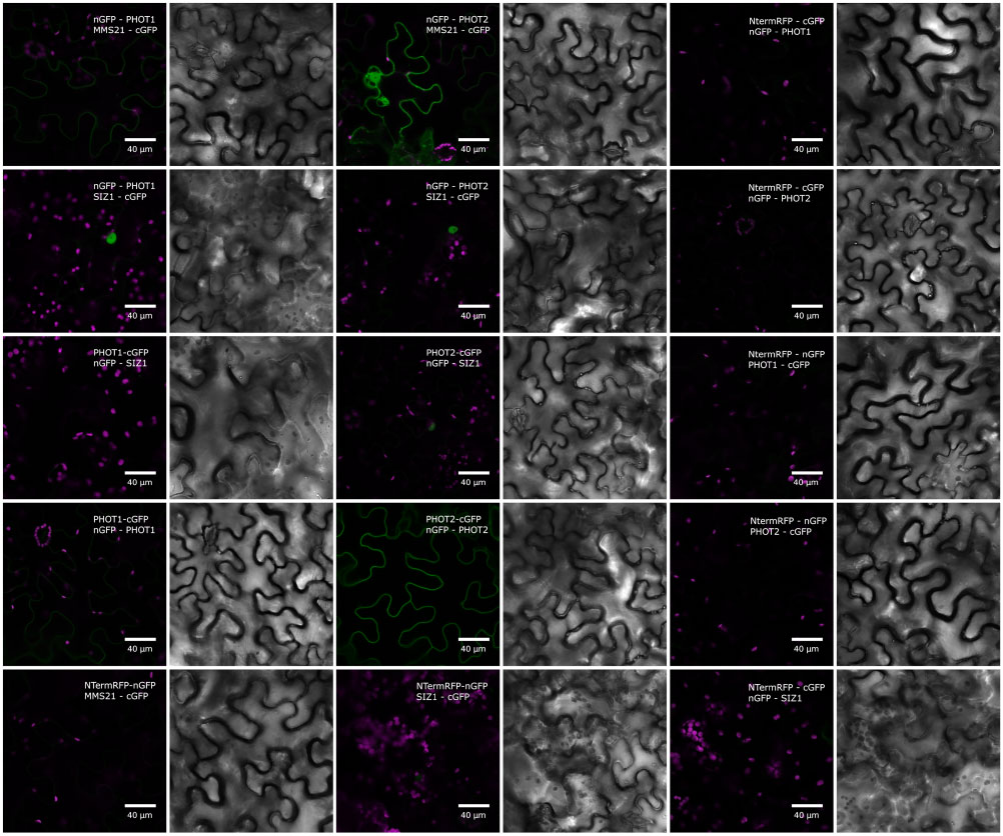
Confocal microscopy images of *N. benthamiana* epidermal cells transiently co-expressing phots fused with the nGFP or cGFP fragment and E3 ligases fused with nGFP or cGFP. Negative control plants expressed the first 150 amino acids from the N-terminal part of the red fluorescent protein fused with the nGFP or cGFP fragment and the tested protein partners fused with appropriate GFP fragments. Leaves co-expressing phots fused with nGFP and with cGFP were used for positive controls, as phots are known to form dimers. Chlorophyll autofluorescence is in magenta and reconstituted GFP fluorescence is in green. Gray scale images show transmitted light. The results represent one of three independent biological replicates.

Bimolecular fluorescence complementation (BiFC) assay was used to investigate the formation of complexes between phots and SUMO proteins or E3 ligases in transiently transformed *Nicotiana benthamiana* leaves. Fluorescence of reconstituted green fluorescent protein (GFP) was observed in the cytoplasm adjacent to the plasma membrane of pavement cells co-transformed with phots fused with C-terminal GFP (cGFP) and either nGFP-SUMO1, nGFP-SUMO2 or nGFP-SUMO3 (Fig. 4). In leaves co-transformed with nGFP-SUMO5 and either PHOT1-cGFP or PHOT2-cGFP, the fluorescence did not exceed the autofluorescence level (Fig. 4). The fluorescence signal indicating the interaction between MMS21-cGFP and nGFP-PHOT1 or nGFP-PHOT2 localized predominantly in the cytoplasm (Fig. 5). In leaves co-transformed with nGFP-PHOT2 and MMS21-cGFP, the fluorescence signal was also visible in the nucleus in cells with high overall signal intensity. Fluorescence localized in the nucleus was observed when SIZ1 and PHOT2 fused with GFP fragments were co-expressed (Fig. 5) in a subset of epidermal cells. No green fluorescence was observed in negative control leaves.

**Fig. 6:**
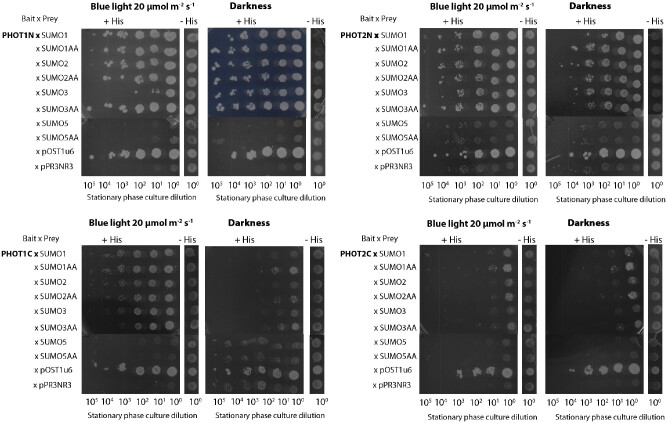
Formation of phot complexes with components of the sumoylation pathway tested with the MYTH assay. N/C-terminal parts of phots were used as baits, SUMO proteins as preys. Native SUMO variants with a diglycine motif at the C-terminus (GG) are marked with no additional letters. The mutated variants, which contain two alanines instead of glycines at the C-terminus, are marked as SUMOAA. Overnight cultures of transformed yeasts were plated on the SC-Leu-Trp (+His) control solid medium or SC-Leu-Trp-His (−His) solid selection medium supplemented with 5 mM 3-aminotriazole (3-AT). The yeast plated on solid media was cultured either in darkness or under blue light (∼20 μmol m^−2^ s^−1^, 455 nm) in 30°C for 4 d. For all bait constructs, co-transformations with positive control pray plasmid (pOST1u6) and empty prey vector (pPR3NR3) were performed to avoid false-negative and false-positive signals respectively, resulting from a nonspecific self-activation. The results represent one of at least three independent biological replicates.

Formation of complexes between phots and components of the sumoylation pathway were confirmed using the split-ubiquitin-based membrane yeast two hybrid (MYTH) system (Fig. 6). Native SUMO and mutated variants containing two alanine residues instead of glycine residues at the C-terminus were used (Okada et al. 2009). The growth of yeast colonies, indicating ubiquitin reconstitution, was observed when the N-terminal part of PHOT1 was expressed with SUMO1, SUMO2 or SUMO3, both in the native (GG) and mutated (AA) variants, irrespective of dark/light conditions. Interactions of those SUMO variants were weak with the PHOT1 whole molecule (Supplementary Fig. S10) or its C-terminal fragment (Fig. 6). The growth of yeast colonies was clearly visible when the N-terminal part of PHOT2 was co-expressed with native (GG) or mutated (AA) variants of SUMO1, SUMO2 or SUMO3 in darkness and light. Negligible interactions were observed for the C-terminal PHOT2 fragment (Fig. 6) and for the whole molecule (Supplementary Fig. S10). N-terminal fragments of PHOT1 and PHOT2 formed weak complexes with SIZ1 and MMS21 ligases (Supplementary Fig. S11).

### Analysis of phot sumoylation using an *in bacteria* system followed by mass spectrometry

**Fig. 7:**
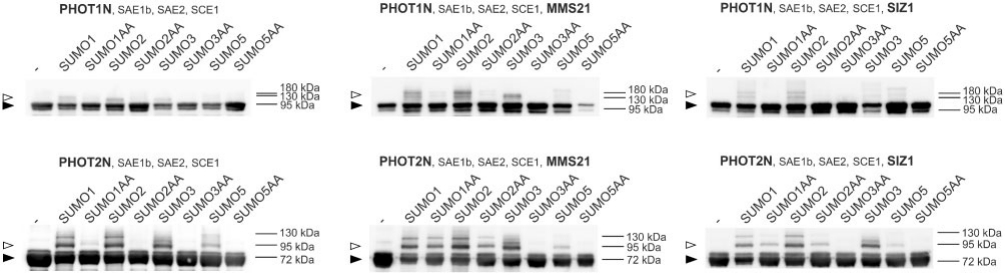
The sumoylation pattern of N-terminal fragments of c-Myc tagged PHOT1 and PHOT2. The Arabidopsis SUMO modification system was reconstituted *in bacteria* by simultaneous overexpression of E1 (AtSAE1b, AtSAE2), E2 (AtSCE1a) and either AtSUMO1, AtSUMO2, AtSUMO3 or AtSUMO5 (native GG or mutated AA variants). In some experimental variants, AtMMS21 or AtSIZ1 were also overexpressed. Phot fragments were purified using Ni-NTA and analyzed by Western blotting, with an anti-c-Myc antibody. In the negative control, only phot fragments were overexpressed in *E. coli* cells. Arrowheads mark positions of non-sumoylated (black) or sumoylated (white) phot fragments. The results are a representative of at least three independent repetitions.

Based on the results of MYTH, N-terminal fragments of phots were chosen for studies using an Arabidopsis sumoylation system reconstituted *in bacteria* according to Okada et al. (2009). E1 (AtSAE1b, AtSAE2) and E2 (AtSCE1a) were co-expressed in *Escherichia coli* together with N-terminal parts of PHOT1 or PHOT2. In some experiments, the SIZ1 or MMS21 E3 ligase was also added (Fig. 7). Without ligases, the N-terminal part of PHOT1 was modified by native SUMO1, SUMO2 and SUMO3, but not by variants bearing GG to AA mutations. In the presence of the MMS21 ligase, a similar sumoylation pattern was visible. When co-expressed with SIZ1, native SUMO1 and SUMO2 were conjugated to the N-terminal PHOT1 fragment. In the absence of ligases, the N-terminal part of PHOT2 was modified by native SUMO1, SUMO2, SUMO3 and SUMO5, and the mutated variants of SUMO proteins were not conjugated. In the presence of either MMS21 or SIZ1, the N-terminal PHOT2 fragment was modified by native and mutated variants of SUMO1 and SUMO2, as well as native SUMO3.

**Fig. 8:**
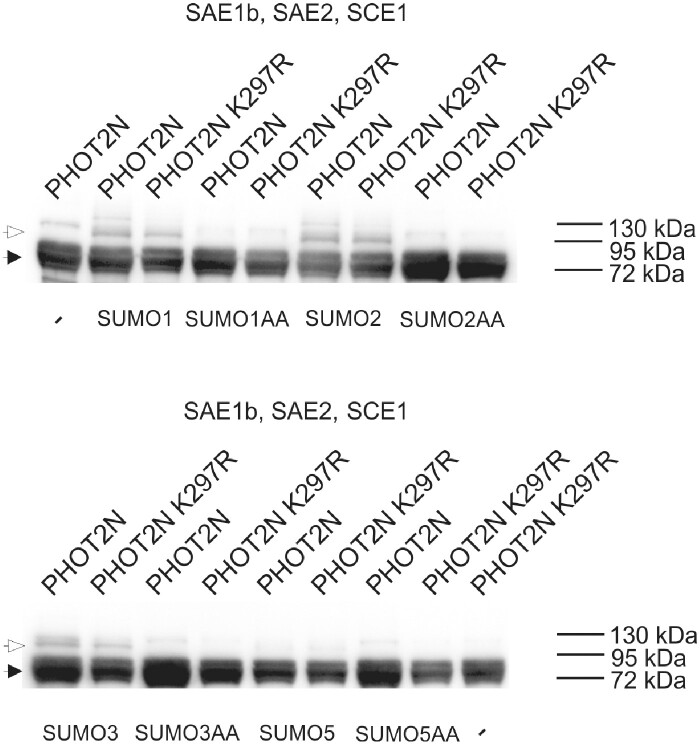
The sumoylation pattern of the N-terminal PHOT2 part bearing the Lys297Arg mutation in comparison to the native form. The Arabidopsis SUMO modification system was reconstituted *in bacteria* by simultaneous overexpression of E1 (AtSAE1b, AtSAE2), E2 (AtSCE1a) and either AtSUMO1, AtSUMO2, AtSUMO3 or AtSUMO5 (native GG or mutated AA variants). PHOT2 fragments were purified using Ni-NTA and analyzed by Western blotting, with an anti-PHOT2 antibody. In the negative control, only the N-terminal part of PHOT2 was overexpressed in *E. coli*. Arrowheads mark positions of non-sumoylated (black) or sumoylated (white) phot fragments.

N-terminal fragments of phots co-expressed with SUMO3 and AtSAE1b, AtSAE2 and AtSCE1a enzymes in *E. coli* were isolated for mass spectrometry (MS)-based sumoylation site mapping. The identified peptides are shown in Table 1, example MS spectra in Supplementary Figs. S12 and S13. In the case of phot1, three residues were identified as sumoylated: Lys 125, 361 and 462. More sites were found for the phot2 molecule, including Lys 32, 43, 51, 56, 65, 79, 90, 125, 197, 283, 267, 297, 333, 516, 545 and 564. In silico analysis of potential sumoylation sites using GPS-SUMO (Zhao et al. 2014) identified three target sites in phot1 and six sites in phot2 (Supplementary Table S1). The online tool JASSA (Beauclair et al. 2015) predicted two high score sumoylation sites in phot1 and three in phot2 (Supplementary Table S2), as well as a SIM motif, consisting of residues 687–690. Both tools predicted that phot2 lysines 79 and 297, identified by MS, are SUMO targets. Thus, Lys 297 was mutated to Arg and analyzed with the Arabidopsis sumoylation system reconstituted *in bacteria*. The Lys 297 to Arg mutation diminished modification of the N-terminal phot2 fragment by native SUMO1, SUMO2, SUMO3 and SUMO5; however, the pattern differed between repetitions (Fig. 8, Supplementary Fig. S14).

**Table 1:** Peptides containing lysine residues modified by SUMO3 identified by MS analysis and the position of modified lysine within phot molecules

Modified peptide sequence	Protein	K position within the protein
ALSESTNLHPFMTKSESDELPK	phot1	361
KGIDLATTLER	phot1	462
AAEWGLVLKTDTK	phot1	125
SQVQESVSNDTMVKPDSSTTPTPGR	phot2	297
KSQVQESVSNDTMVKPDSSTTPTPGR	phot2	283; 297
VKPDSSTTPTPGR	phot2	197
TAEWGLSAVKPDSGDDGISFK	phot2	79; 90
TAEWGLSAVKPDSGDDGISFKLSSEVER	phot2	79; 90
ETHGSTSSSSKPPLDGNNK	phot2	43; 51
ETHGSTSSSSKPPLDGNNKGSSSK	phot2	43; 51
SLEIFNPSSGKETHGSTSSSSKPPLDGNNK	phot2	43; 32
ELPDANTRPEDLWAAHSKPVYPLPHNK	phot2	545
LVKATATNVDEAVR	phot2	516
VSTPTGSKLK	phot2	333
EKALDSITEVVQTIR	phot2	267
QKEKALDSITEVVQTIR	phot2	267
GSSSKWMEFQDSAK	phot2	56; 65
WMEFQDSAKITER	phot2	65
KIQASGETVGL	phot2	564

### Analysis of phot2 sumoylation in planta

**Fig. 9:**
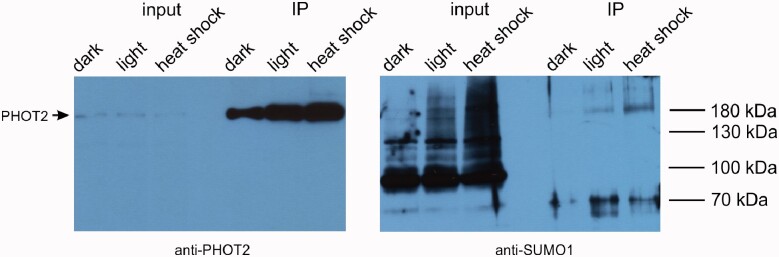
Sumoylation of phot2 *in planta.* Arabidopsis plants expressing PHOT2-GFP were subjected to three types of treatments: adapted overnight to darkness, exposed to photoperiodic light for 1 h or subjected to a heat shock of 40°C for 15 min after 1 h photoperiodic light. PHOT2-GFP was immunoprecipitated from leaf extracts using an anti-GFP antibody (GFP-Trap), followed by a Western Blot with anti-PHOT2 antibody (left) or anti-SUMO1 antibody (right). Sumoylation is indicated by the appearance of high molecular mass bands in the immunoprecipitated (IP) samples.

To confirm phot2 sumoylation *in planta*, a protein immunoprecipitation experiment was performed using an Arabidopis line expressing PHOT2-GFP. Dark-adapted plants were kept in darkness or exposed to photoperiodic light for 1 h with or without an additional heat shock of 40°C for 15 min, a condition that elicits protein sumoylation by SUMO1 and SUMO2 (Kurepa et al. 2003). We observed that sumoylation of input samples was enhanced after heat shock treatment (Fig. 9). Additional bands of high molecular weight, over 180 kDa, were detected by the anti-SUMO1 antibody in fractions containing immunoprecipitated PHOT2 from plants exposed to light or light followed by heat shock treatments. This indicates sumoylation of the photoreceptor. The corresponding band was also observed in the immunoprecipitated fraction from heat treated plants labeled with anti-PHOT2 antibodies (Fig. 9).

## Discussion

Two phot controlled responses, phototropism in seedlings and chloroplast movements in mature leaves, seem to be sensitive to impaired sumoylation. Enhanced chloroplast accumulation responses to continuous light and to pulses were observed in the *siz1* mutant as compared to the wild type (Figs. 1B, 2C, D). The *siz1* mutant shows a dwarf phenotype (Supplementary Fig. S1, Lin et al. 2016). Despite the smaller cell size, its leaf structure is similar to the wild type (Miura et al. 2010). Dark transmittance levels, which reflect chloroplast distribution before the light treatment and can influence their light-induced relocation, were comparable in leaves of all investigated lines (Supplementary Figs. S15–S17). Also, our microscopic observations indicate that the dark positioning of chloroplasts in palisade cells is not affected by the mutation in the *SIZ1* gene (Fig. 1E). The *phot1siz1* mutant showed stronger accumulation amplitudes to both continuous and pulse blue light than *phot1* plants (Figs. 1C, 2E, F). By contrast, the magnitude of chloroplast responses observed in *phot2siz1* and *phot2* plants was similar (Figs. 1D, 2G, H), even though all *siz1* background lines are dwarfs (Supplementary Fig. S1). Thus, enhanced chloroplast accumulation characteristic for *siz1* and *phot1siz1* seems to depend on both the presence of phot2 and sumoylation defects caused by the *siz1* mutation. At this stage, it is not possible to determine if this is the effect of altered sumoylation of phot2 itself or of proteins either involved in signal transduction to chloroplast movements or being essential for this process.

**Fig. 10:**
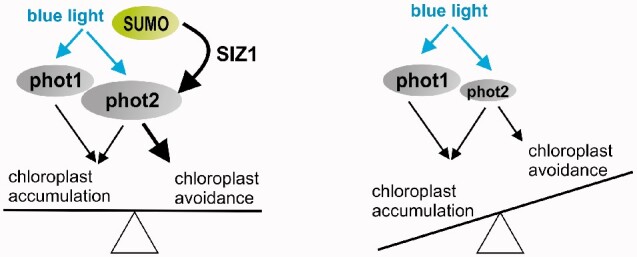
A model of the modulatory role of sumoylation in the control of chloroplast movements.

In Arabidopsis rosette leaves treated with blue light of 120 µmol m^−2^ s^−1^, the levels of phot1 and phot2 proteins were lower in *siz1* plants than in the wild type, while amounts of their transcripts were similar (Supplementary Fig. S8). One of the possible ways in which sumoylation affects protein activity is by increasing its stability, as is the case for Flowering Locus C (Kwak et al. 2016). In an analogous manner, phots might be stabilized after blue light treatment through the E3 activity of SIZ1. Our observation that sumoylation of phot2 is induced by light in Arabidopsis leaves (Fig. 9) is consistent with this model. A recent study reported lower phosphorylation levels of phot1 in the *siz1* mutant than in wild-type Arabidopsis plants (Nukarinen et al. 2017). How these effects are linked to the activity of photoreceptors in eliciting chloroplast movements remains to be determined. Chloroplast movements can be dependent either on absolute amounts of each phot or the ratio of these photoreceptors. Chloroplast avoidance velocity is dependent on the amount of phot2 (Kagawa and Wada 2004), therefore shifting the equilibrium toward accumulation may result from lower protein levels. Phots can interact with each other forming homo- and heterodimers and this fine tunes chloroplast movements (Sztatelman et al. 2016). When the amount of phot2 changes, the homo-to-heterodimer ratio will be altered, further influencing the equilibrium between chloroplast accumulation and avoidance. A graphical model of the effect of sumoylation on chloroplast movements is shown in Fig. 10.

In very weak blue light of 0.01 µmol m^−2^ s^−1^, phototropic bending was diminished in *mms21*, *phot2mms21* and *phot2siz1* mutants (Fig. 3A, B); however, we observed morphological differences between etiolated seedlings grown *in vitro*. The difference in phototropic bending between wild-type and *mms21* plants may result from growth retardation of the mutant, as it exhibits a dwarf phenotype (Huang et al. 2009, Ishida et al. 2009) (Fig. 3C). On the other hand, the double *phot2mms21* mutant displayed a partially restored morphology of seedlings. The growth of *phot2mms21* and *phot2siz1* (Fig. 3C) was intermediate between the wild type and the dwarf phenotypes of *siz1* (Lin et al. 2016) or *mms21* (Huang et al. 2009), respectively. This suggests that the *PHOT2* gene is to some extent epistatic to E3 ligase genes. The differences in phototropic bending were not directly correlated with changes in phot expression levels (Supplementary Fig. S9A–D).

The influence of sumoylation pathway disruption on responses triggered by phots could be explained by sumoylation of phots. Phots were predicted to undergo modification by SUMO in Elrouby and Coupland (2010). Phot1 and phot2 interacted with SUMO1, SUMO2 and SUMO3 in planta, as shown by BiFC (Fig. 4). Yeast two hybrid assays indicated that these complexes formed mainly through phot N-terminal parts (Fig. 6). Results from experiments using Arabidopsis sumoylation system reconstituted *in bacteria* suggest that N-terminal phot fragments could undergo sumoylation (Fig. 7). Several bands were observed when the N-terminal part of PHOT2 was modified by SUMO1 and SUMO2 (Fig. 7), pointing to the possibility of poly- (addition of SUMO chains) or multi-sumoylation (addition of single-SUMO molecules in several places on the target). Both SUMO1 and SUMO2 may form poly-SUMO chains, as they bear surface accessible sumoylation motifs, while SUMO3 is added only as single molecules (Colby et al. 2006). Addition of the MMS21 or SIZ1 ligase to the system resulted in a loss of specificity, i.e. the N-terminal fragment of PHOT2 was modified both with native SUMO1GG and SUMO2GG as well as with mutated SUMO1AA and SUMO2AA. This was not reported before and may be limited to the model system overexpressing all proteins tested. Interestingly, *SUM4* and *SUM6* genes, whose expression *in planta* has not been confirmed yet, contain an SG motif at their potential conjugation site (TAIR, www.arabidopsis.org). MS analysis showed that numerous lysine residues of the N-terminal PHOT2 fragment were modified by SUMO3 when co-expressed with proteins of the Arabidopsis sumoylation pathway in *E. coli.* Lys 297 was dominant among the identified residues. Mutational analysis indicated that this residue was modified by SUMO1, 2, 3 and 5 (Fig. 8). Lys 297 lies in a phot2 region, which is highly phosphorylated after blue light irradiation (Christie et al. 2015). The sequence around Lys 297 in the PHOT2 molecule resembles a phosphorylation-dependent sumoylation motif (PDSM). In animals, phosphorylation is linked with sumoylation through PDSM (CKXEXXSP, where S, serine; P, proline), consisting of a SUMO consensus sequence and a proline-directed phosphorylation site (Hietakangas et al. 2006). This points to an interesting possibility that sumoylation of phot2 depends on its phosphorylation and thus may be regulated by light. Our results from the PHOT2-GFP immunoprecipitation experiment further support this hypothesis, as phot2 seems to be modified by SUMO1 in light but not in dark conditions (Fig. 9). As discussed above, sumoylation can influence phot stability. Phot2 is constantly degraded in darkness, but not under blue light illumination (Aggarwal et al. 2014). It cannot be excluded that light-induced sumoylation of phot2 (Fig. 9) is responsible for this effect. Phot2 sumoylation was also enhanced by a heat shock (Fig. 9), possibly influencing signaling. In tobacco, a rapid heat shock treatment (25–40–25°C) inhibits chloroplast responses (Frolec et al. 2010).

Our results suggest that SUMO ligases affect responses triggered by phots: chloroplast accumulation is favored in the *siz1* mutant and reduced phototropic bending is observed in the *mms21* mutant. Components of the signaling pathways or phots themselves may be the targets for SUMO. The N-terminal part of phot2 has more potential sites modified by SUMO than the corresponding part of phot1, as identified by MS analysis. One of them is Lys 297, which was identified as a SUMO target. It lies in a heavily phosphorylated region of the photoreceptor. Further studies are needed to elucidate the physiological role of phot2 sumoylation at this site.

## Materials and Methods

### Plants and growth conditions

The following *A. thaliana* lines were used in this study: wild-type Col-0, *sum1* SAIL_296_C12, *sum2* SALK_129775C, *sum3* SALK_123673C, *sum5* SALK_085812C*, siz1-3* SALK_034008, *mms21-1* SAIL_77_G06 (Huang et al. 2009), *phot1* SALK_088841 (Lehmann et al. 2011), *phot2 npl1-1* (Jarillo et al. 2001) and a transgenic line expressing PHOT1*::*PHOT2-GFP *(*a kind gift of J.M. Christie, Hart et al. 2019). T-DNA mutant lines were purchased from Nottingham Arabidopsis Stock Centre or were kindly gifted by the indicated authors. The double mutants, *Atphot1siz1-3*, *Atphot1mms21-1*, *Atphot2siz1-3*, *Atphot2mms21-1*, were selected from crosses. The homozygosity of lines was confirmed using Phire Plant Direct PCR Master Mix (Thermo Fisher Scientific, Waltham, MA, USA) and primers listed in Supplementary Table S3. For experiments using seedlings, seeds were surface sterilized with 70% ethanol for 5 min and 50% Ace (commercial bleach) for 5 min, washed three times in distilled, sterile water and sown on solid MS medium with vitamins and 3% sucrose. For experiments with leaves, 5-week-old plants were used. Seeds were sown in Jiffy-7 pots (Jiffy Products International AS, Stange, Norway) and left at 4°C for 2 d. Then, plants were transferred to a growth chamber (MLR 350H, Sanyo, Osaka, Japan) at 23°C, 80% relative humidity, with a photoperiod of 10-h light and 14-h darkness, at 70 μmol m^−2^ s^−1^ of light supplied by fluorescent lamps (FL40SS.W/37, Sanyo).

### Assessment of chloroplast movements

Chloroplast movements were assessed with the photometric method, as described in Gabryś et al. (2017). In this approach, chloroplast movements are followed by measurements of changes in leaf transmittance. Plants were dark-adapted for at least 16 h before the measurement. Responses to 0.1, 0.2, 1, 2, 10 and 20 s blue light pulses of 120 µmol m^−2^ s^−1^ and continuous blue light of increasing intensity 0.4, 1.6, 4, 20, 40, 80 and 120 µmol m^−2^ s^−1^ (50 min for each light regime) were determined. Photometric curves were analyzed using a custom-written Mathematica (Wolfram Research, Champaign, IL, USA) package. The amplitudes (Δ*T*) with respect to the dark level and the maximal velocities (d*T*/d*t*) of transmittance changes were calculated. Chloroplast arrangements in mesophyll cells were analyzed as in Hermanowicz et al. (2019), by assessing chlorophyll autofluorescence under a confocal microscope. Arabidopsis leaves were kept in darkness (mock irradiation) or irradiated with blue light (LED 460 nm, 1 W, epiLED, Wrocław, Poland) of 1.6 or 120 µmol m^−2^ s^−1^ for 50 min. Images were recorded with the objective LD LCI Plan-Apochromat 25×/0.8. Chlorophyll fluorescence was excited with the 633 nm He–Ne laser and emission was collected in the range of 661–721 nm. Maximum intensity projections were calculated from Z-stacks, which spanned whole depth of the epidermis and palisade parenchyma, starting from the leaf upper surface.

### Measurements of phototropic curvature

Arabidopsis wild-type and mutant seeds were sown on square agar plates, kept at 4°C for 2 d and irradiated with white light for 2 h. Seedlings were grown in darkness on vertically positioned plates for 3 d. Phototropic bending experiment was performed as in Zeidler (2016). The bending angle was measured as shown in Fig. 3A. Seedlings were irradiated for 12 h with blue light (Luxeon LED, LXHL-PR09, Lumileds, Schipol, Netherlands) of either 0.01 or 5 µmol m^−2^ s^−1^. Irradiance (light intensity) was measured with a LI-190R sensor (Licor Biosciences, Lincoln, NE, USA).

### Determination of phot expression

Phot expression analysis at mRNA and protein levels was performed in five biological replicates for Arabidopsis etiolated seedlings and mature leaves. Leaves of 4-week-old plants were dark-adapted overnight and irradiated with blue light of 120 µmol m^−2^ s^−1^ (Luxeon LED, LXHL-PR09) for 3 h. Dark-adapted leaves collected at the same time served as a control. Each sample contained material from two plants. Three-day-old etiolated seedlings were illuminated with blue light (Luxeon LED, LXHL-PR09) of either 0.01 or 5 µmol m^−2^ s^−1^ for 12 h. Etiolated seedlings kept in darkness were collected at the same time as the control. Each sample contained material from 50 seedlings. Samples were frozen in liquid nitrogen immediately after treatments.

RNA isolation and real-time PCR were performed as in Łabuz et al. (2012), except for RNA reverse transcription performed with oligo (dT) primers. Primer sequences are listed in Łabuz et al. (2012) for *PHOT1* and *PHOT2* and in Czechowski et al. (2005) for reference genes: *UBC*, *PDF2* and *SAND*. Each sample was quantified in three technical replicates. The mean value of Ct for samples from all experimental groups quantified simultaneously was subtracted from individual Ct values, for the purpose of inter-run calibration. Expression levels were then normalized using factors calculated by geNorm v3.4 (Vandesompele et al. 2002).

Proteins were extracted as in Sakamoto and Briggs (2002). Samples were homogenized, weighed and adjusted to equal mass. SDS–PAGE and Western Blot were performed as in Sztatelman et al. (2016). Membranes were incubated with anti-PHOT2 antibodies (AS10721, Agrisera, Vännäs, Sweden) at a dilution of 1:5,000 or anti-PHOT1 (AS10720, Agrisera) antibodies at 1:300 (a purified fraction)overnight in 4°C (see Łabuz et al. 2015). Secondary antibodies *(*goat anti-rabbit horseradish peroxidase-conjugated IgG, Agrisera), diluted 1:25,000, were incubated at room temperature for 1 h. Signal detection was performed with a Clarity Western ECL Blotting Substrate (Bio-Rad, Hercules, CA, USA) by the BioSpectrum Imaging System (UVP, Analytik Jena US, USA). Intensities of the chemiluminescent signal were normalized to actin levels in each sample. For this, membranes were stripped with Restore Plus Western Blot Stripping Buffer (Thermo Fisher Scientific) and probed with anti-actin antibody (AS132640, Agrisera), diluted 1:2,500, at room temperature for 1 h, followed by secondary antibody incubation and ECL detection. Densitometric quantification was performed using ImageJ.

### BiFC

MultiSite Gateway system (Thermo Fisher Scientific) was used to prepare constructs for BiFC analysis. PHOT1 and PHOT2 constructs were described in Sztatelman et al. (2016), SUMO3 in Strzalka et al. (2012). SIZ1, MMS21, SUMO1, SUMO2 and SUMO5 constructs were cloned into pDONR221 with the Easy-A High Fidelity polymerase (Agilent Technologies, Santa Clara, CA, USA) and confirmed by sequencing. Primers and vectors used for cloning are listed in Supplementary Tables S4 and S5. Destination vectors were described by Karimi et al. (2007). The cGFP fragment fused to the first 150 amino acids from the N-terminal part of the red fluorescent protein was used for the negative BiFC control (Strzalka et al. 2015). Transient transformation of *N. benthamiana* leaves was performed as described in Aggarwal et al. (2014). Microscopic observations were performed 2 d after transformation, using the Axio Observer.Z1 inverted microscope (Carl Zeiss, Jena, Germany) equipped with the LSM 880 confocal module. Plan-Neofluar 40× objective was used with oil immersion. Argon laser line of 488 nm was used for the excitation of GFP and chlorophyll. Emission within the range of 493–573 nm was recorded as the green channel. Emission in the range of 651–721 nm was recorded as the red channel.

### Split-ubiquitin-based MYTH system

Protein interactions were tested in yeast using the split-ubiquitin-based MYTH system (MoBiTec, Goettingen, Germany), with introduced Gateway cloning sequences (Strzalka et al. 2015). Bait and prey vectors with full-length phots or their N- or C-terminal domains were described in Sztatelman et al. (2016). The N-terminal PHOT1 fragment (PHOT1N) consisted of amino acids 1–619, and the N-terminal PHOT2 fragment (PHOT2N) consisted of amino acids 1–574. The C-terminal part of PHOT1 contained amino acids 620–996, and the C-terminal part of PHOT2 consisted of amino acids 575–915. pPR3_N_Gateway prey and pDHB1_Gateway bait vectors, described in Strzalka et al. (2015), and pDONR221 with SIZ1, MMS21 and SUMO1, SUMO2, SUMO3, SUMO5 in GG or AA forms were used to prepare the destination constructs. All primers are listed in Supplementary Table S4. Yeast transformation was performed as in Strzalka et al. (2015). For scoring interactions, agar plates with transformed yeast were kept at 30°C either in darkness or under continuous blue light (**∼**20 µmol m^−2^ s^−1^, 455 nm 1 W LEDs, epiLED) for 4 d. Each experiment was repeated three times.

### In bacteria analysis of sumoylation

Reconstitution of the sumoylation system in *E. coli* was performed as in Okada et al. (2009). BL21(DE3) cells were transformed with plasmids pACYCDUET-1:AtE1, encoding AtSAE1b and AtSAE2, and pCDFDUET-1:AtE2:SUMO, encoding AtSCE1a and one of the variants (GG or AA) of SUMO1, 2, 3 or 5 (Okada et al. 2009). In addition, bacteria containing either pET15b:MMS21 or pMAL:SIZ1 were prepared. The pMAL:SIZ1 vector was a kind gift of Nam-Hai Chua, The Rockefeller University, New York, USA. The pET15b:MMS21 vector was prepared by amplifying the *MMS21* sequence with primers 5′GGAATTCCATATGGCGTCGGCGTCCTCGTCTG3′ and 5′CGGGATCCCTAATCTTCATCCACATCTTCTG3′. The sequence was cloned into pET15b using the NdeI and BamHI sites. Bacteria were transformed with either pET28a:c-Myc-6xHis-PHOT1N (amino acids 1–662) or pET28a:c-Myc-6xHis-PHOT2N (amino acids 1–574). pET28a was modified by inserting the sequence coding for c-Myc into the NcoI site. The c-Myc sequence was obtained by annealing overlapping oligonucleotides 5′CATGGAACAGAAACTGATCTCTGAAGAAGACCTGGC3′ and 5′CATGGCCAGGTCTTCTTCAGAGATCAGTTTCTGTTCC3′. PHOT1N amplified with 5′TACTGGATCCATGGAACCAACAGAAAAACCATCG3′ and 5′TACTAAGCTTATGCTTCAAACCAATCGGTTCACC3′ was cloned into the modified pET28acMyc vector using BamHI and NdeI restriction sites. PHOT2N was amplified using primers 5′TACTGTCGACTTATAGTCCCACTGTTTCTCCACTC3′ and 5′TACTCATATGGAACAGAAACTGATCTCTGAAGAAGACCTGATGGAGAGGCCAAGAGCCC3′, which contained the c-Myc coding sequence and cloned into standard pET28a using NdeI and SalI sites. To introduce the Lys 297 to Arg mutation in the PHOT2N fragment, Quik Change II XL Site-Directed Mutagenesis Kit, 200521 (Agilent Technologies), was used with primers 5′GCAATGACACTATGGTAAGACCTGATAGTTCTACTACACC3′ and 5′GGTGTAGTAGAACTATCAGGTCTTACCATAGTGTCATTGC3′. Bacteria were grown at 37°C in LB medium containing chloramphenicol (25 mg l^−1^), kanamycin (50 mg l^−1^) and spectinomycin (100 mg l^−1^). Ampicillin (100 mg l^−1^) was also added when pET15b:MMS21 or pMAL:SIZ1 was present. After reaching OD_600_ of 0.6, temperature was decreased to 20°C, protein expression was induced in cultures with 0.2 mM IPTG (isopropyl-β-d-thiogalactopyranoside), and bacteria were grown for 20 h. Further procedures were performed at 4°C. Cells were centrifuged at 5,000× *g* for 15 min, resuspended in 1 ml of protein extraction buffer (50 mM NaH_2_PO_4_, 300 mM NaCl and 10 mM imidazole, pH 8.0, protease inhibitor cocktail), sonicated for 5 min, with 5 s pulses and 10 s intervals and centrifuged at 35,000× *g* for 20 min. Twenty microliters of Ni-NTA agarose (Qiagen, Hilden, Germany) was added to the cell lysate and incubated for 1 h with constant rotation. Unbound proteins were removed by washing three times with 1 ml of extraction buffer containing 20 mM imidazole for 5 min. Bound proteins were eluted with 20 µl of extraction buffer containing 250 mM imidazole, mixed with SDS–PAGE loading buffer and denatured at 100°C for 5 min. Samples were separated by SDS–PAGE using 12% gels and transferred onto a PVDF membrane (0.2 µm) (Merck, Darmstadt, Germany). The membrane was blocked in 5% milk in PBS (Phosphate buffered saline) with 0.5% Tween 20 for 30 min and incubated for 2 h with a monoclonal anti-c-Myc antibody (clone 9E10), diluted 1:20,000 (Merck). Then, it was washed three times with 5% milk in PBS with 0.5% Tween 20 for 5 min and incubated for 1 h with a goat anti-mouse IgG–horseradish peroxidase conjugate antibody, diluted at 1:20,000 (Merck). After three washes in PBS with 0.5% Tween 20, chemiluminescent detection was performed using a Clarity Western ECL Blotting Substrate (Bio-Rad) by the BioSpectrum Imaging System.

### In silico and statistical analysis

*In silico* analysis of potential sumoylation sites in phot molecules was performed using GPS-SUMO (Zhao et al. 2014) and JASSA (Beauclair et al. 2015). Statistical calculations were performed using the R software. The mRNA and protein levels were log-transformed before statistical analysis; other measurements were not transformed. Significance of the effects of the plant line and light conditions was analyzed with one- or two-way ANOVA, followed by Dunnett’s test, used for pairwise comparisons between mutant lines and the control lines. *phot1* line was treated as a control for *phot1mms21* and *phot1siz1*, *phot2* for *phot2mms21* and *phot2siz1* and the wild type for other lines. The *P-*values reported in the text and figures are adjusted for multiple comparison.

### Proteomic analysis—MS and protein identification

N-terminal phot fragments co-expressed with SUMO3 and AtSAE1b, AtSAE2 and AtSCE1a (Okada et al. 2009) were purified from *E. coli*. Cells were resuspended in 1 ml of c-Myc protein extraction buffer [50 mM Tris, 150 mM NaCl, 0.5 mM EDTA, pH 7.5, supplemented with 25 mg l^−1^ lysozyme and 2× protease inhibitor cocktail (Complete, Merck)], sonicated for 30 s, with 0.5 s pulses in 0.5 s intervals and centrifuged at 30,000× *g* for 20 min. Twenty-five microliters of Myc-Trap Magnetic Agarose (ChromoTek, Planegg-Martinsried, Germany) suspended in 250 µl of c-Myc protein extraction buffer was added to the cell lysate and incubated for 90 min with constant rotation. Unbound proteins were removed by washing four times with 1 ml of c-Myc protein extraction buffer without lysozyme and protease inhibitors for 5 min. Beads were suspended in SDS–PAGE loading buffer and denatured at 95°C for 5 min. Samples were separated by SDS–PAGE using 4–20% gradient Mini-PROTEAN TGX Stain-Free Precast Gel (Bio-Rad) and stained with colloidal Coomassie. Protein bands corresponding to sumoylated phot fragments were excised from the gel and subjected to standard procedure of trypsin digestion. Proteins were reduced with 10 mM DTT for 30 min at 56°C, alkylated with iodoacetamide in darkness for 45 min at room temperature and digested overnight with 10 ng µl^−1^ trypsin. The resulting peptide mixtures were concentrated and desalted on a RP-C18 pre-column (Waters, Milford, MA, USA). Further peptide separation was achieved on a nano-Ultra Performance Liquid Chromatography RP-C18 column (Waters, BEH130 C18 column, 75 µm i.d., 250-mm long) using a 160-min linear acetonitrile gradient in the presence of 0.1% formic acid. Column outlet was directly coupled to the ion source of the Orbitrap Elit mass spectrometer (Thermo Electron Corp., San Jose, CA, USA), working in the regime of data dependent MS to MS/MS switch. A blank run ensuring absence of cross-contamination from previous samples preceded each analysis. The acquired MS/MS data were pre-processed with Mascot Distiller software (v. 2.6, MatrixScience, London, UK). A search was performed with the Mascot Search Engine (MatrixScience, Mascot Server 2.5) against the TAIR10 database (35,386 sequences; 14,482,855 residues). To reduce mass errors, the peptide and fragment mass tolerance settings were established separately for individual LC–MS/MS runs after a measured mass recalibration (Malinowska et al. 2012). The rest of search parameters was as follows: enzyme, semiTrypsin, missed cleavages, fixed modifications, Carbamidomethyl (C), variable modifications, Oxidation (M), Sumo (K) (a short peptide fragment of SUMO3 after tryptic digestion that remains linked with lysine—AMSGG), instrument and HCD. Protein identification was performed using the Mascot search engine with the probability based algorithm. The expected value threshold of 0.05 was used for analysis, which means that all peptide identifications had <1 in 20 chance of being a random match.

### Protein immunoprecipitation

For the analysis of protein sumoylation in planta, 5-week-old *A. thaliana* plants expressing PHOT2-GFP (Hart et al. 2019) were used. Plants were dark-adapted overnight and subjected to three types of treatments: 1 h in darkness, 1 h exposure to photoperiodic light with or without heat shock of 40°C for 15 min. Leaves were snap frozen in liquid nitrogen and ground to fine powder. Leaf material was suspended 1:1 in ice cold Pi-RIPA buffer [10 mM sodium phosphate pH 7.5, 150 mM NaCl, 0,5 mM EDTA, 0,1% SDS, 1% Triton X-100, 1% deoxycholate, 20 mM NEM, protease inhibitor cocktail (Complete), 1 mM Phenylmethylsulfonyl fluoride, PMSF]. Samples were rotated for 20 min at 4°C, followed by centrifugation at 16,000× *g*, 20 min, 4°C. Supernatants were diluted 1:1 with an ice cold dilution buffer (50 mM Tris, 150 mM NaCl, 0.5 mM EDTA), mixed with 20 µl of GFP-Trap Agarose (ChromoTek) and incubated for 60 min at 4°C with constant rotation. Unbound proteins were removed by washing once with 1 ml of a 1:1 mixture of Pi-RIPA and dilution buffer and twice with 1 ml of 10 mM Tris, 150 mM NaCl and 0.5 mM EDTA for 5 min each. Beads were suspended in SDS–PAGE loading buffer and denatured at 95°C for 5 min. Samples were split in two parts, separated by SDS–PAGE in 8% gels and transferred to a PVDF membrane using a wet transfer system (Bio-Rad) followed by immunodetection with anti-PHOT2 (as described above) or anti-SUMO1 (Abcam, Cambridge, UK) antibodies. The membrane was blocked in 5% milk in PBS with 0.1% Tween 20 for 1 h and incubated overnight at 4°C with anti-SUMO1 antibody, diluted at 1:2,000. Then, it was washed three times with 5% milk in PBS with 0.1% Tween 20 for 5 min and incubated for 1 h with a goat anti-rabbit IgG–horseradish peroxidase conjugate antibody, diluted at 1:50,000 (Agrisera). After three washes in PBS with 0.1% Tween 20, chemiluminescent detection was performed using a SuperSignal^™^ West Pico PLUS Chemiluminescent Substrate (Thermo Fisher Scientific) and a Carestream MXBE film (Carestream Health, Rochester, NY, USA).

## Supplementary Data

Supplementary data are available at PCP online.

## Funding

The Polish National Science Centre [Grant No. UMO-2011/01/B/NZ3/02160 to H.G., UMO-2017/25/B/NZ3/01080 to J.L. and UMO-2016/22/E/NZ3/00326 to A.K.B.]. Confocal microscopy was carried out thanks to the Polish Innovation Economy Operational Program from the European Regional Development Fund [Contract No. POIG.02.01.00-12-167/08, project Małopolska Centre of Biotechnology]. The open-access publication of this article was funded by the Priority Research Area BioSunder the program ‘‘Excellence Initiative –Research University” at the Jagiellonian University in Krakow.

## Disclosures

No conflicts of interest are declared.

## Supplementary Material

pcab027_Supp
