# Supplementary material for: Phototropin Interactions with SUMO Proteins
Source: Plant Cell Physiol. 2021 Feb 17;62(4):693–707. doi: 10.1093/pcp/pcab027 (PMC8462379; doi:10.1093/pcp/pcab027)
Supplement: pcab027_Supp [file pcab027_supp.zip › pcp-2020-e-00456-File012.docx]

**Supplementary information**


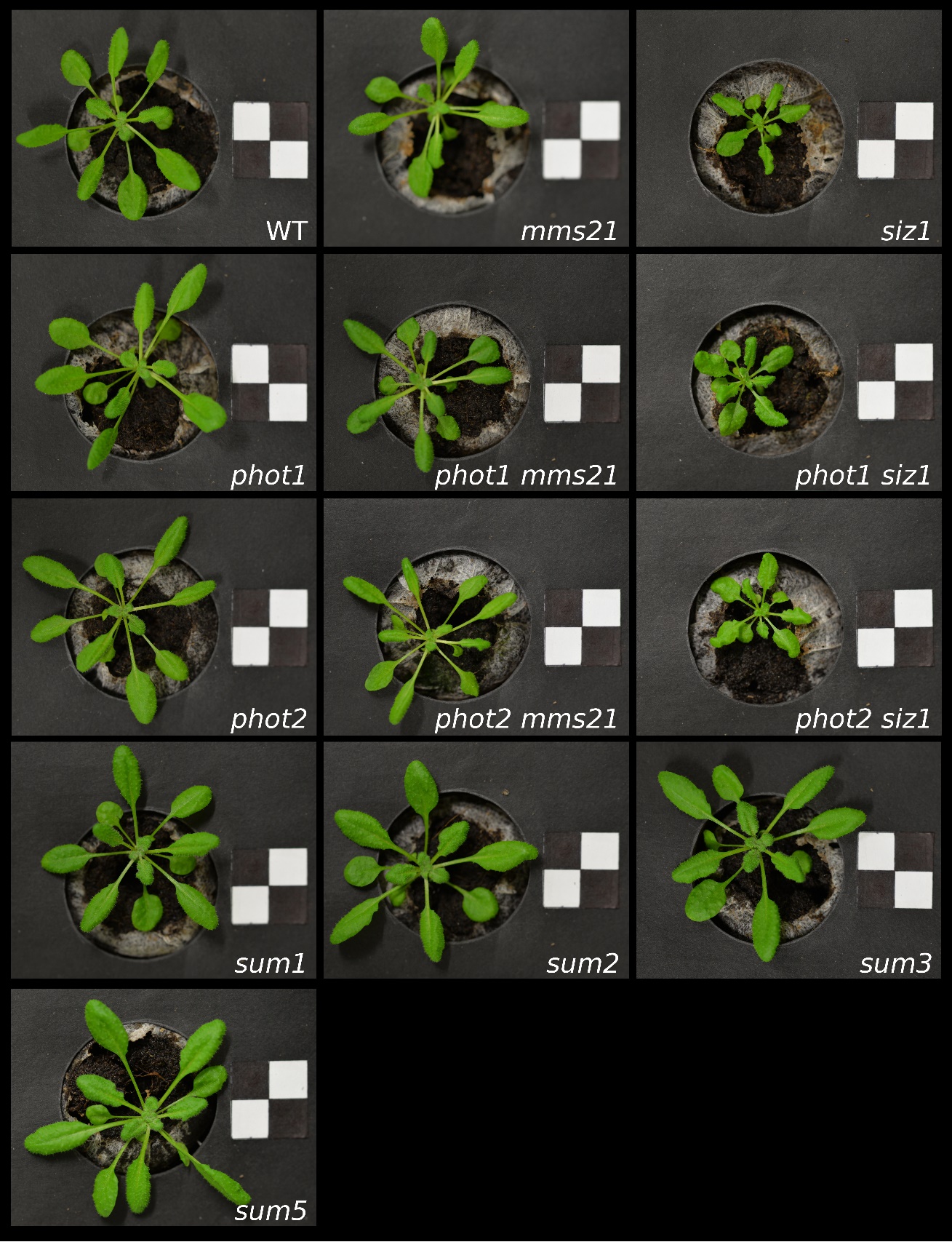


Fig. S1. The phenotype of 4-week old *Arabidopsis* mutants of the sumoylation pathway, grown in the photoperiod of 10 h light and 14 h darkness, at 70 μmol·m^-2^·s^-1^. White rectangles are 1 cm per 1 cm.


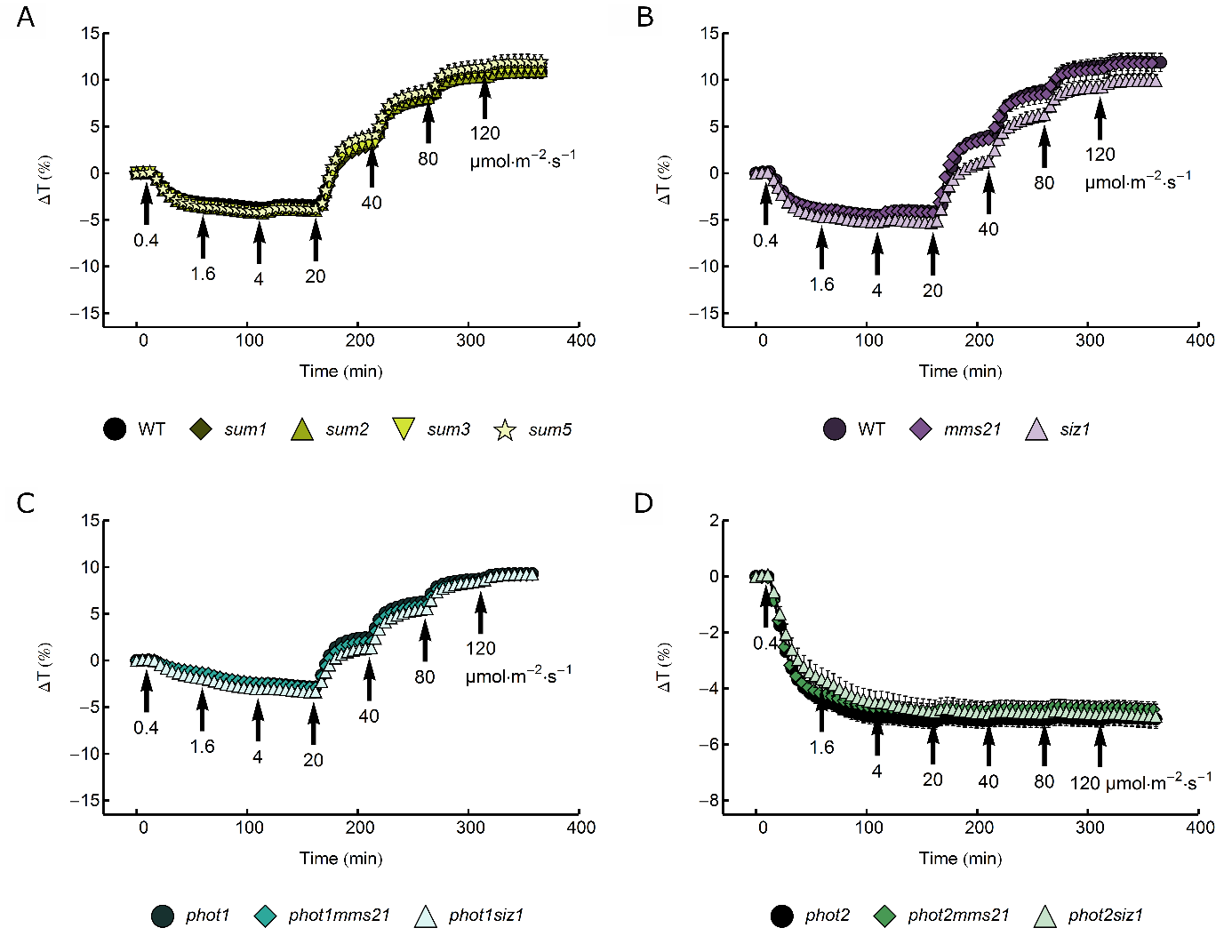


Fig. S2. Chloroplast movements in response to continuous blue light of increasing light intensity in A: *sum* mutants, B: *siz1* and *mms21* mutants, C: double *phot1siz1* and *phot1mms21* mutants, D: double *phot2siz1* and *phot2mms21* mutants. Wild-type *Arabidopsis* (WT) is the control line in A and B, in C *phot1* and in D *phot2* act as the control. Time course of changes in red light transmittance were recorded after applying blue light of increasing intensity 0.4, 1.6, 4, 20, 40, 80, 120 µmol m^−2^ s^−1^. Each data point is an average of at least 9 measurements.


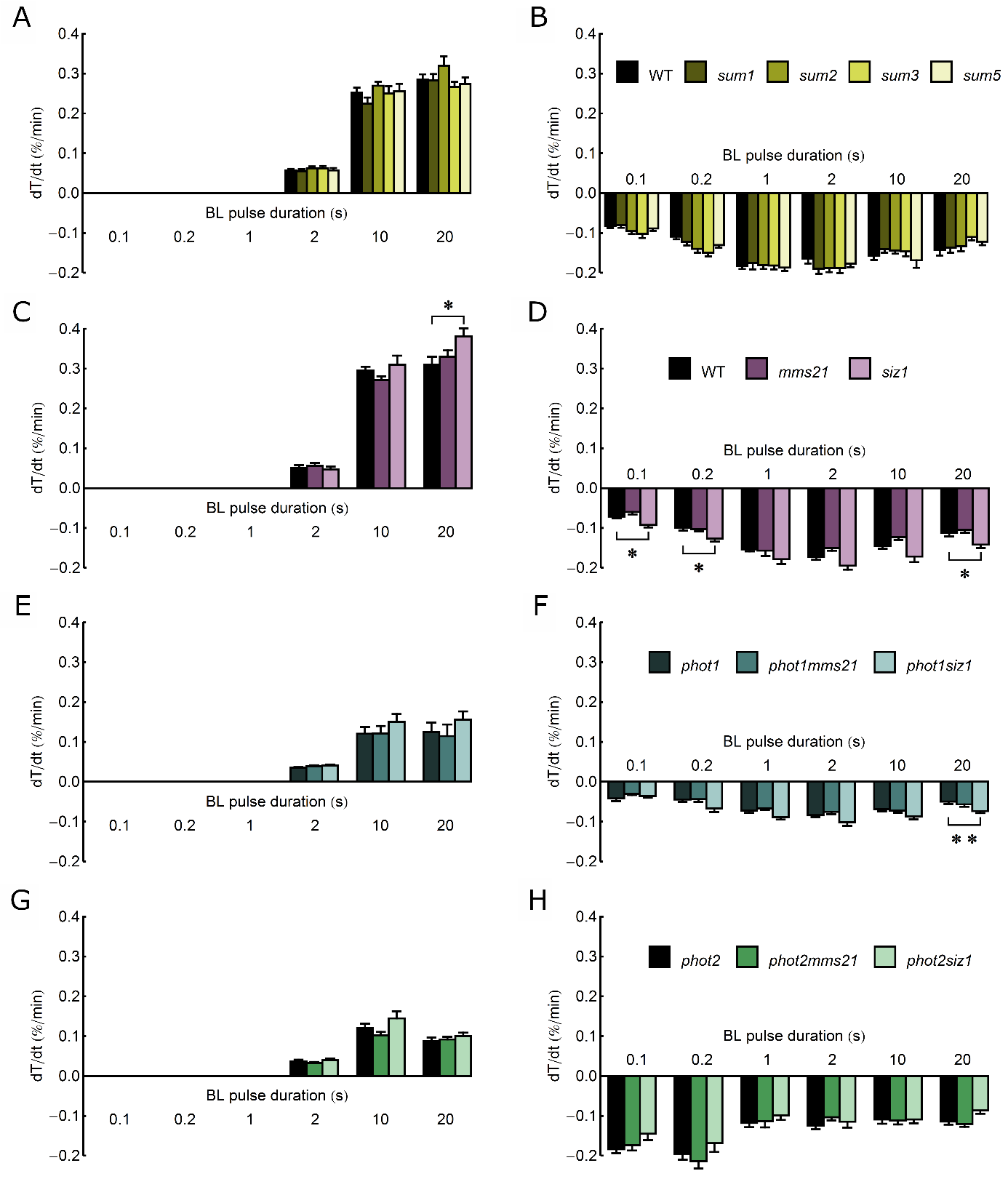


Fig. S3. Rates of transmittance changes dT/dt due to chloroplast movements triggered by 0.1 – 20 s long blue light pulses of 120 µmol·m^-2^·s^-1^ in A,B: *sum* mutants; C,D: *siz1* and *mms21*; E,F: *phot1siz1* and *phot1mms21*; G,H: *phot2siz1* and *phot2mms21*. The values were calculated for the transient A,C,E,G: avoidance and B,D,F,H: accumulation responses. Asterisks indicate statistically significant differences between mutant lines and the control (*phot1* for *phot1mms21* and *phot1siz1*, *phot2* for *phot2mms21* and *phot2siz1*, wild type (WT) for other lines), as tested with the Dunnett’s test (*P=0.01–0.05; **P=0.001–0.01, ***P<0.001). Error bars = SE.


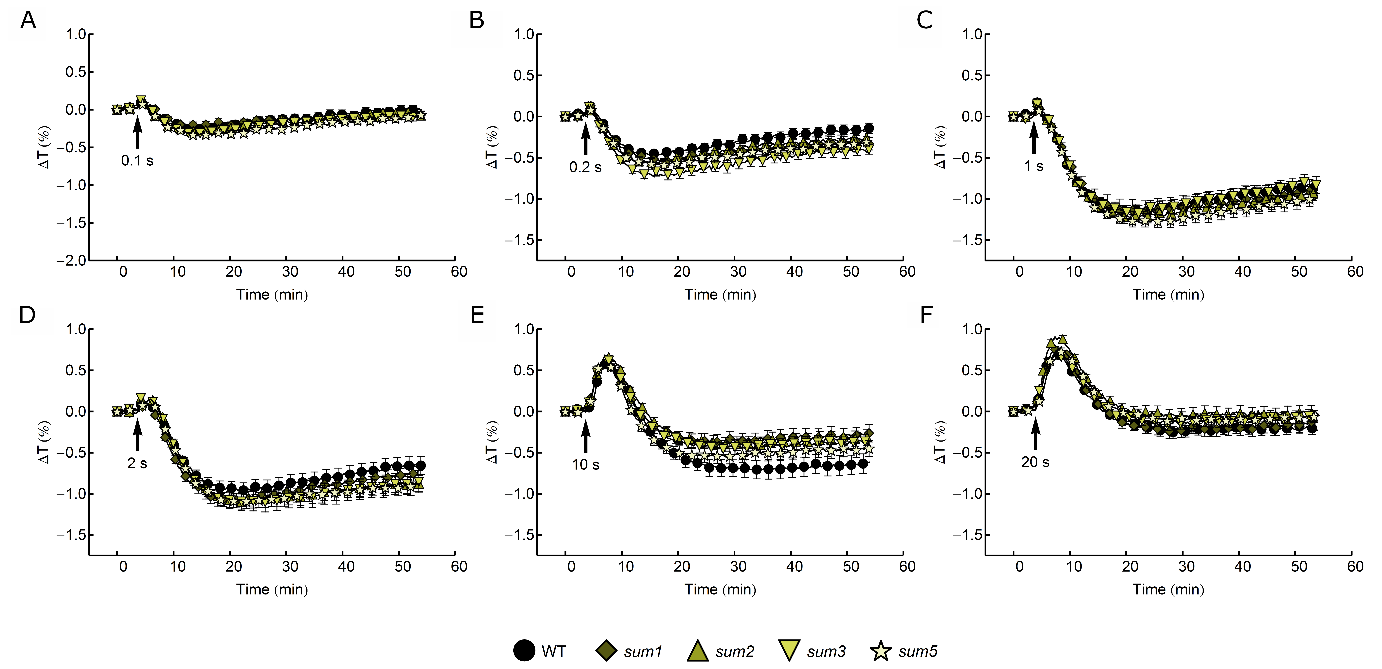


Fig. S4. Chloroplast movements in response to strong blue light pulses in wild-type *Arabidopsis* and *sum* mutants. Time course of changes in red light transmittance were recorded before and after a blue light pulse of 120 µmol m^−2^ s^−1^ and duration specified in the figure. Each data point is an average of at least 18 measurements. Error bars show the SE.


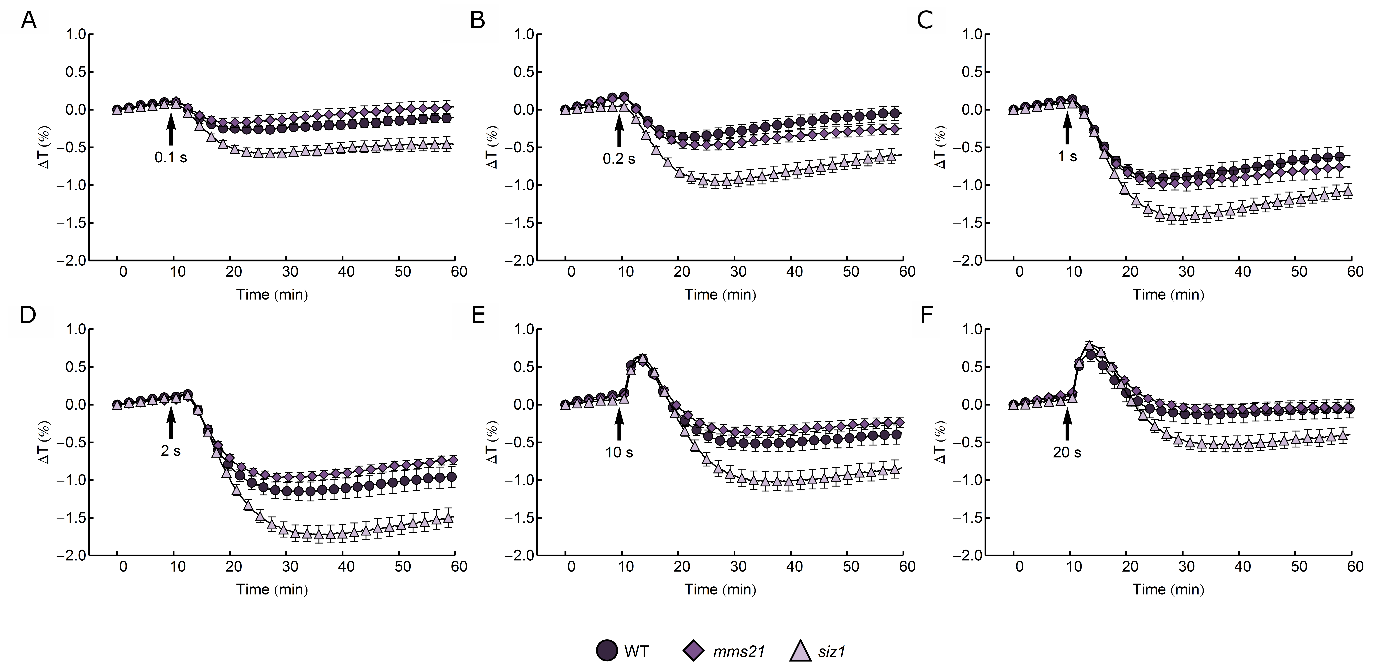


Fig. S5. Chloroplast movements in response to strong blue light pulses in wild-type *Arabidopsis* and *mms21* and *siz1* mutants. Time course of changes in red light transmittance were recorded before and after a blue light pulse of 120 µmol m^−2^ s^−1^ and duration specified in the figure. Each data point is an average of at least 13 measurements. Error bars show the SE.


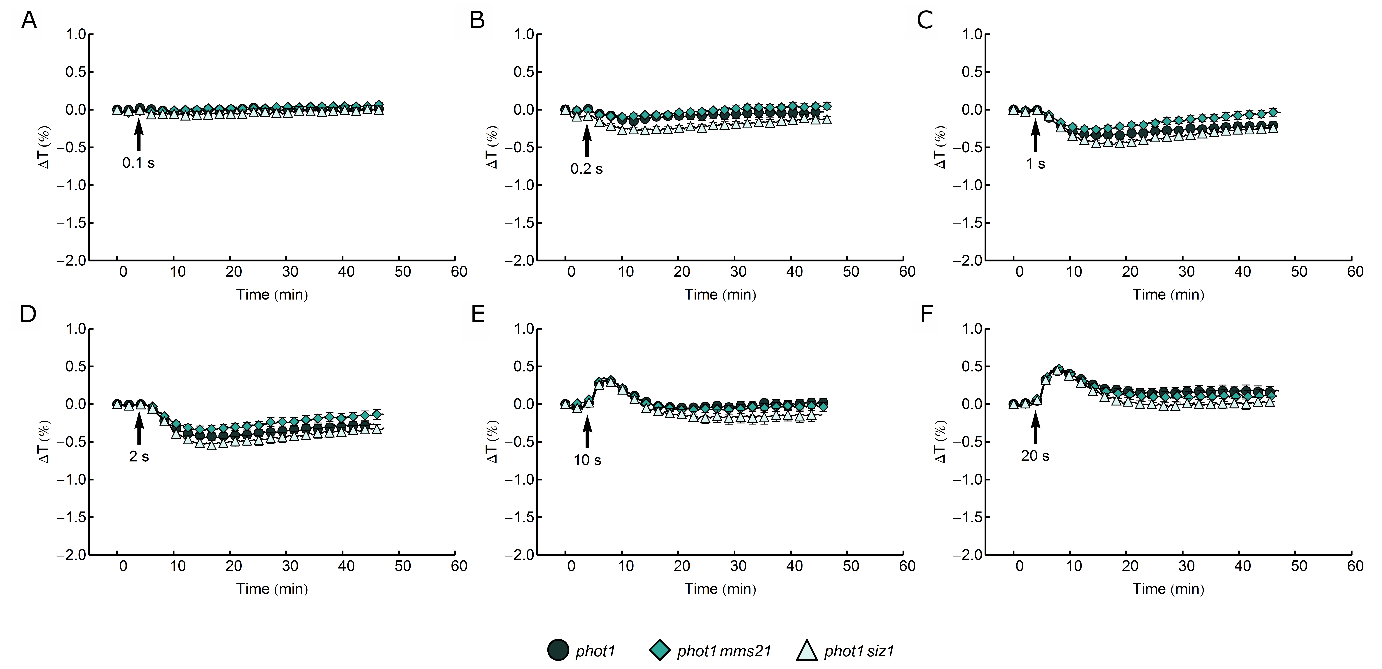


Fig. S6. Chloroplast movements in response to strong blue light pulses in *Arabidopsis phot1* and *phot1mms21* and *phot1siz1* mutants. Time course of changes in red light transmittance were recorded before and after a blue light pulse of 120 µmol m^−2^ s^−1^ and duration specified in the figure. Each data point is an average of at least 14 measurements. Error bars show the SE.


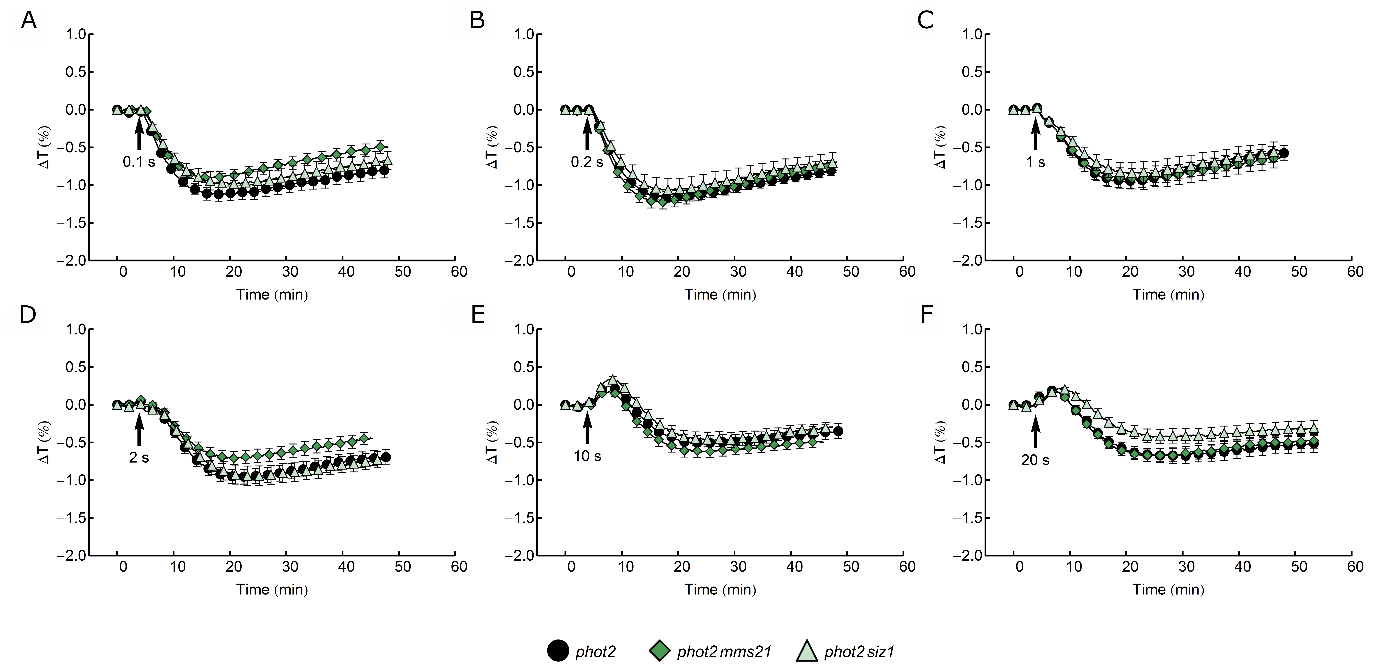


Fig. S7. Chloroplast movements in response to strong blue light pulses in *Arabidopsis* *phot2* and *phot2mms21* and *phot2siz1* mutants. Time course of changes in red light transmittance were recorded before and after a blue light pulse of 120 µmol m^−2^ s^−1^ and duration specified in the figure. Each data point is an average of at least 13 measurements. Error bars show the SE.


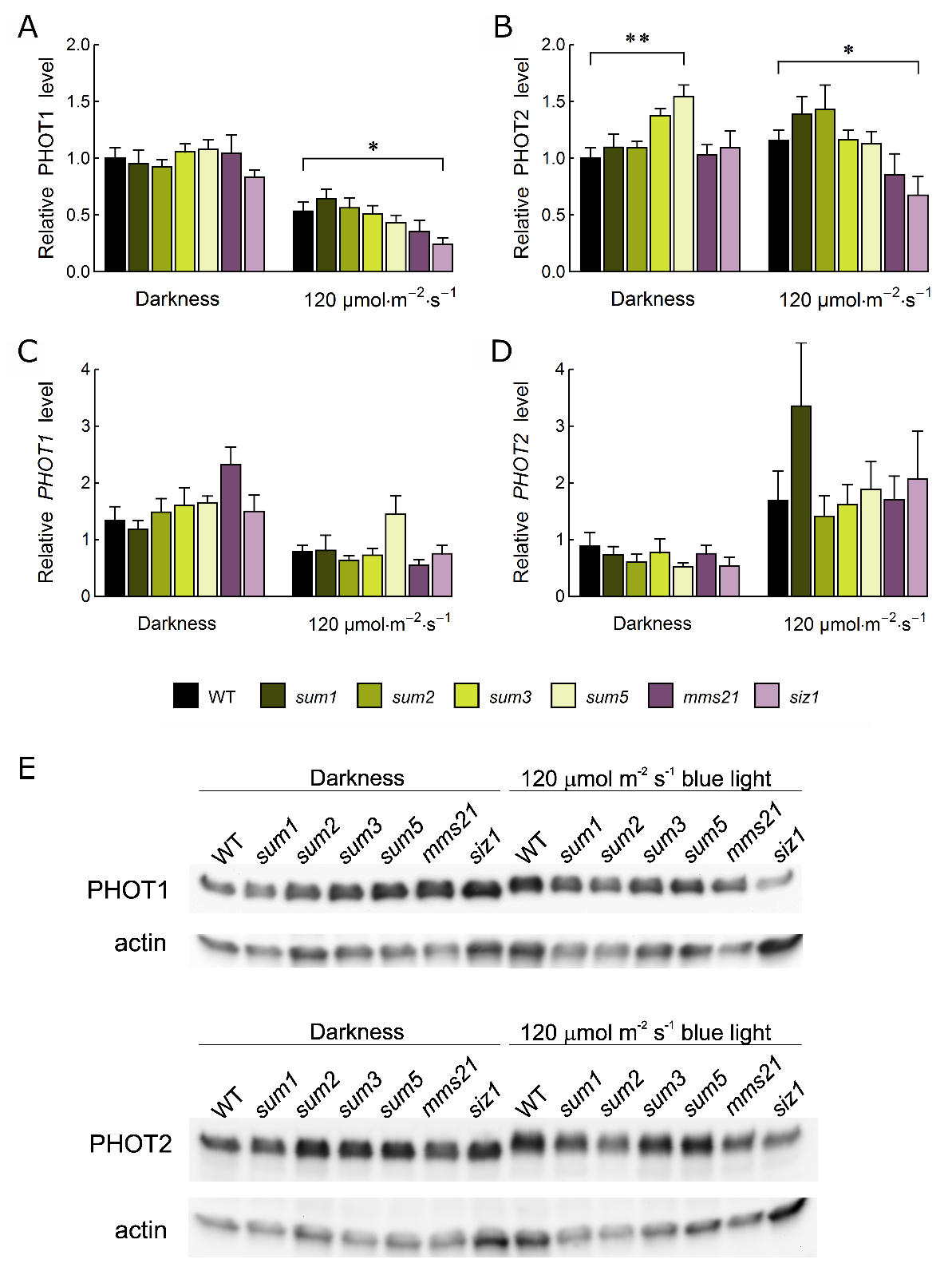


Fig. S8. The relative expression at A,B: protein and C,D: mRNA levels of phot1 and phot2 in rosette leaves of 4-week-old *Arabidopsis* mutants of the sumoylation pathway. Dark adapted plants were irradiated with blue light of 120 µmol·m^-2^·s^-1^ for 3 h or kept in darkness. The levels in the wild type were set to 1. Each bar corresponds to an average of five biological replicates. Asterisks indicate statistically significant differences between mutant lines and wild type, as tested with the Dunnett’s test on log – transformed data (*P=0.01–0.05; **P=0.001–0.01, ***P<0.001). Error bars = SE. E: Example Western Blot used for densitometric analysis.


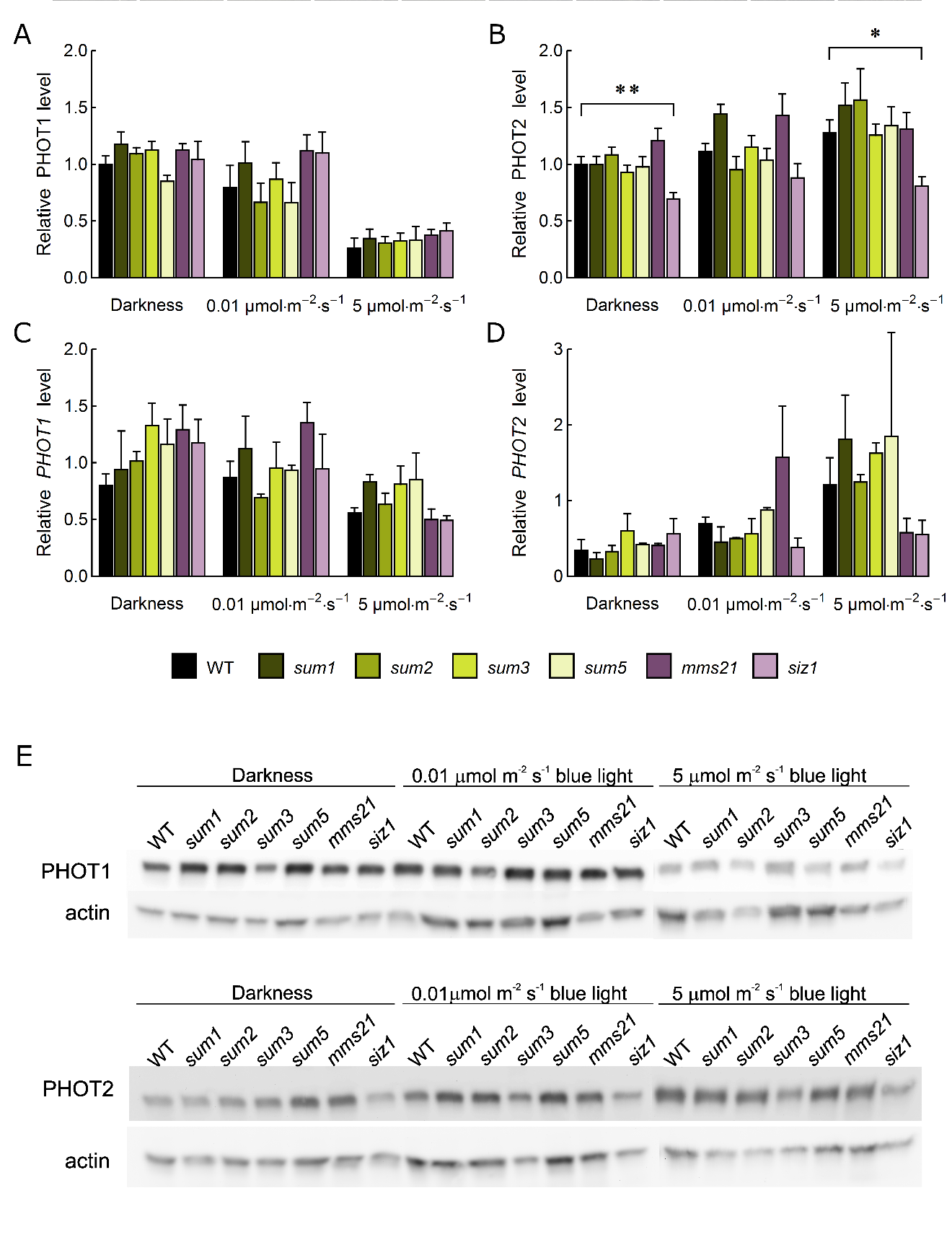


Fig. S9. The relative expression at A,B: protein and C,D: mRNA levels of phot1and phot2 in 3-day-old etiolated seedlings after 12 h-long treatment with blue light of 0.01 or 5 µmol·m^-2^·s^-1^. The levels in the wild type were set to 1. Each bar corresponds to an average of five biological replicates. Asterisks indicate statistically significant differences between mutant lines and wild type, as tested with the Dunnett’s test on log – transformed data (*P=0.01–0.05; **P=0.001–0.01, ***P<0.001). Error bars = SE. E: Example Western Blot used for densitometric analysis.


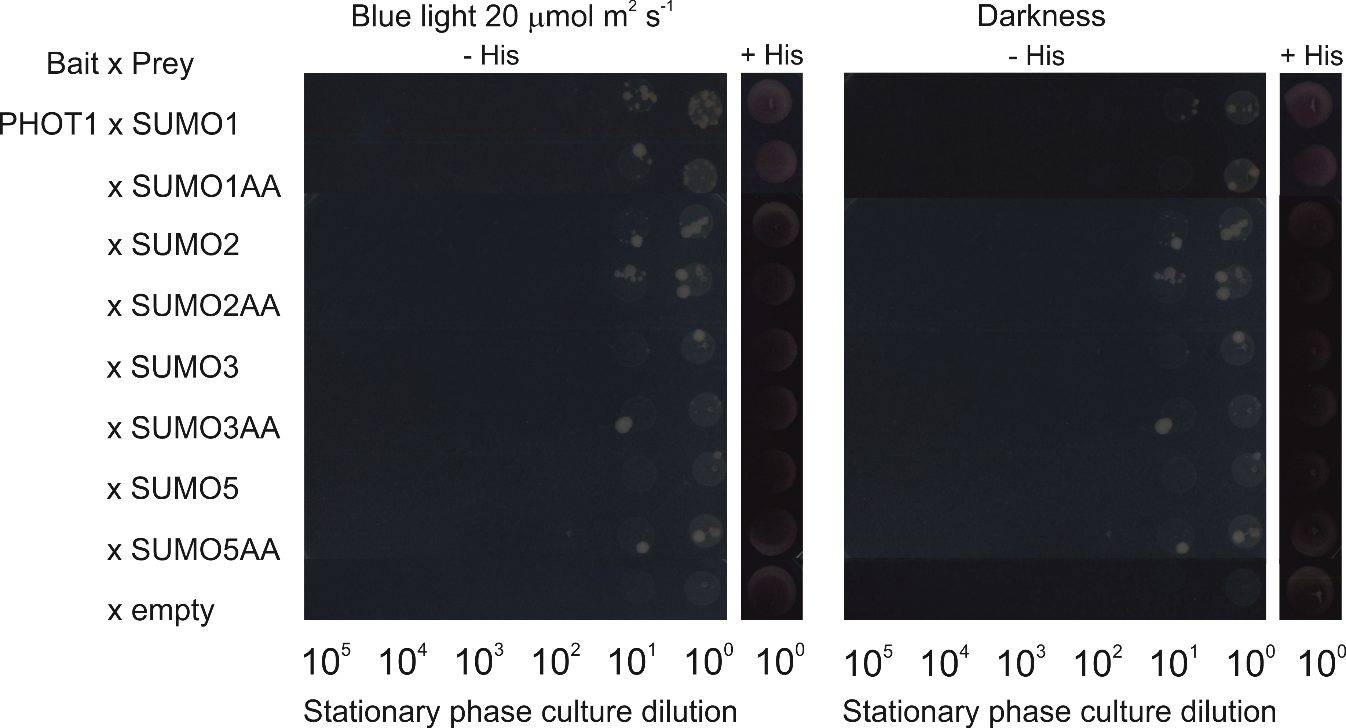

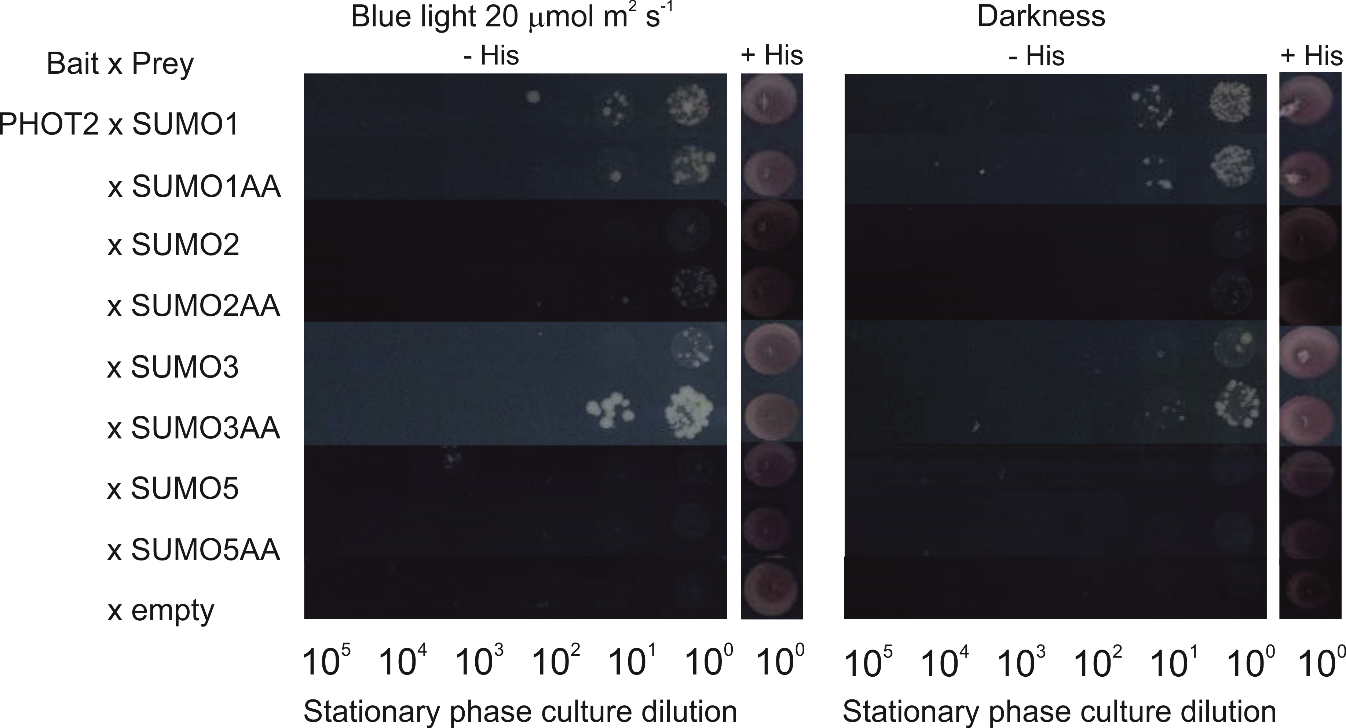


Fig. S10. Interactions of whole phototropins with SUMO tested with the MYTH assay. full-length phototropins were used as baits and SUMO as preys. Overnight cultures of transformed yeasts were plated on the SC-Leu-Trp (+His) control solid medium or SC-Leu-Trp-His (-His) solid selection medium supplemented with 5 mM 3-aminotriazole (3-AT). The yeast plated on solid media were cultured either in darkness or under blue light (~20 μmol m^-2^ s^-1^, 470 nm) in 30°C for 4 days. For all bait/prey constructs, a co-transformation with empty prey/bait vectors was performed to avoid false-positive signals resulting from a non-specific self-activation. The results represent one of at least three independent biological replicates.


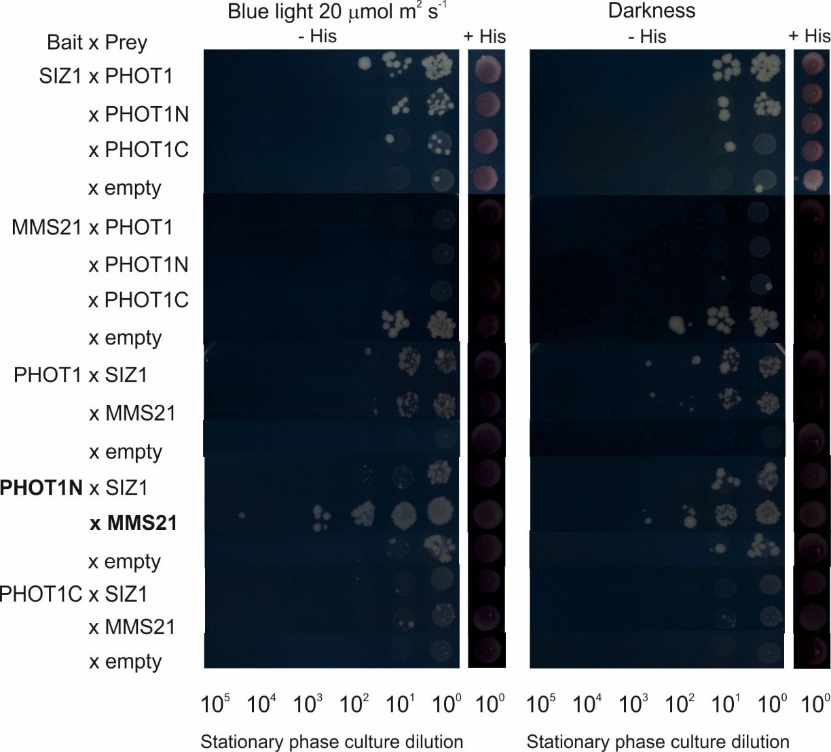

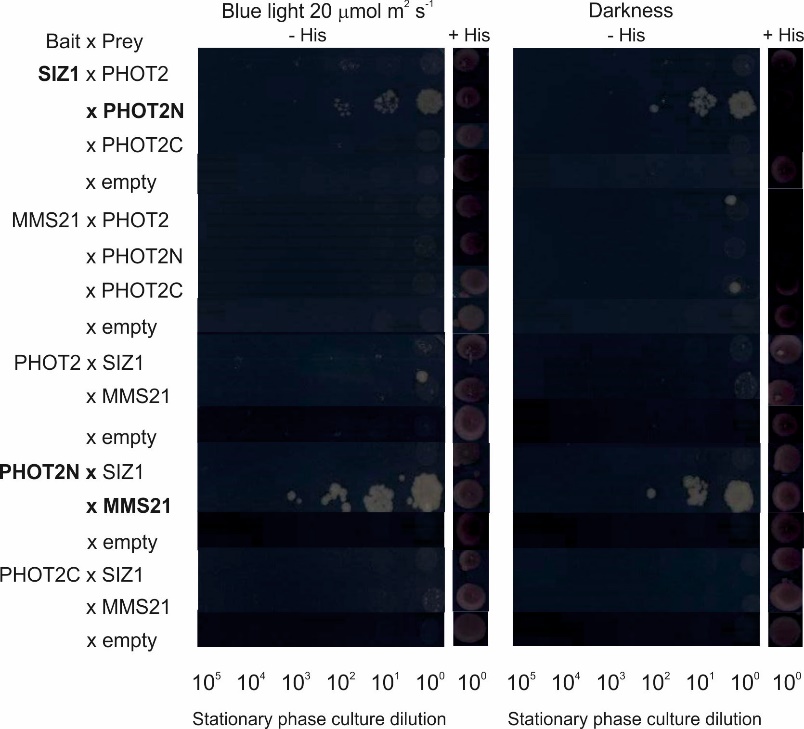


Fig. S11. Interactions of phototropins with SIZ1 or MMS21 ligases tested with the MYTH assay. E3 ligases, full-length phototropins and their N/C-terminal parts were used as baits and preys. Overnight cultures of transformed yeasts were plated on the SC-Leu-Trp (+His) control solid medium or SC-Leu-Trp-His (-His) solid selection medium supplemented with 5 mM 3-aminotriazole (3-AT). The yeast plated on solid media were cultured either in darkness or under blue light (~20 μmol m^-2^ s^-1^, 460 nm) in 30°C for 4 days. For all bait/prey constructs, a co-transformation with empty prey/bait vectors was performed to avoid false-positive signals resulting from a non-specific self-activation. The results represent one of at least three independent biological replicates.


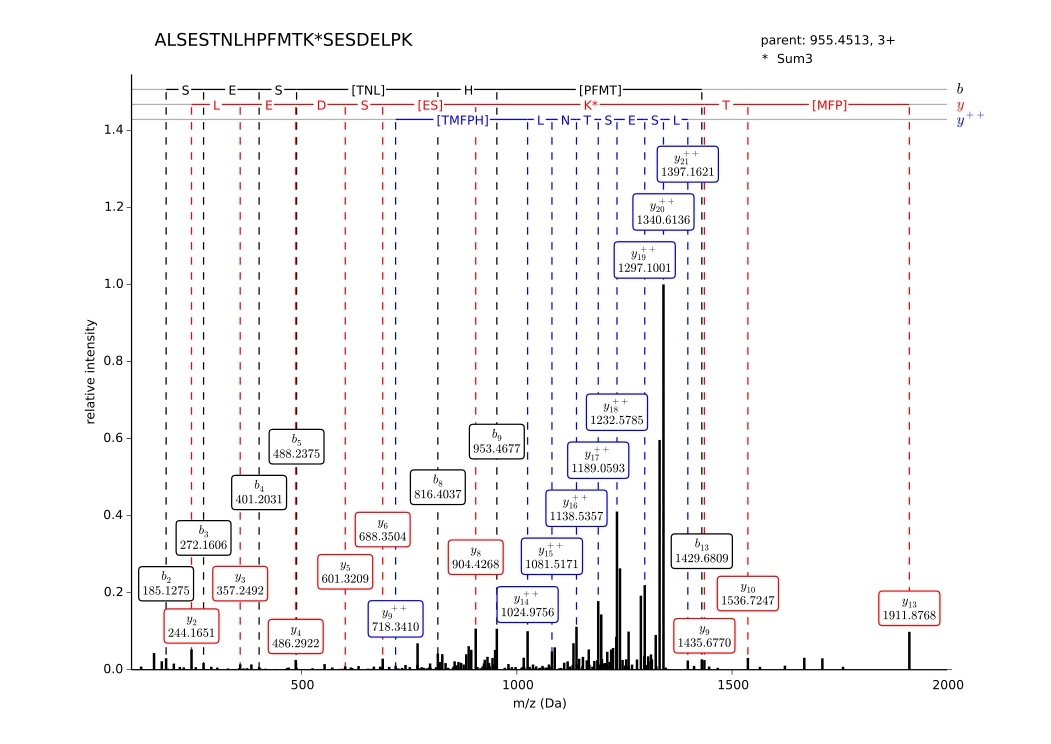


Fig. S12. Example MS spectra of tryptic digests of phot1 for sumoylation site mapping. SUMO3 modified phot1 peptides are linked via lysine residue with a short fragment of SUMO3 – AMSGG.


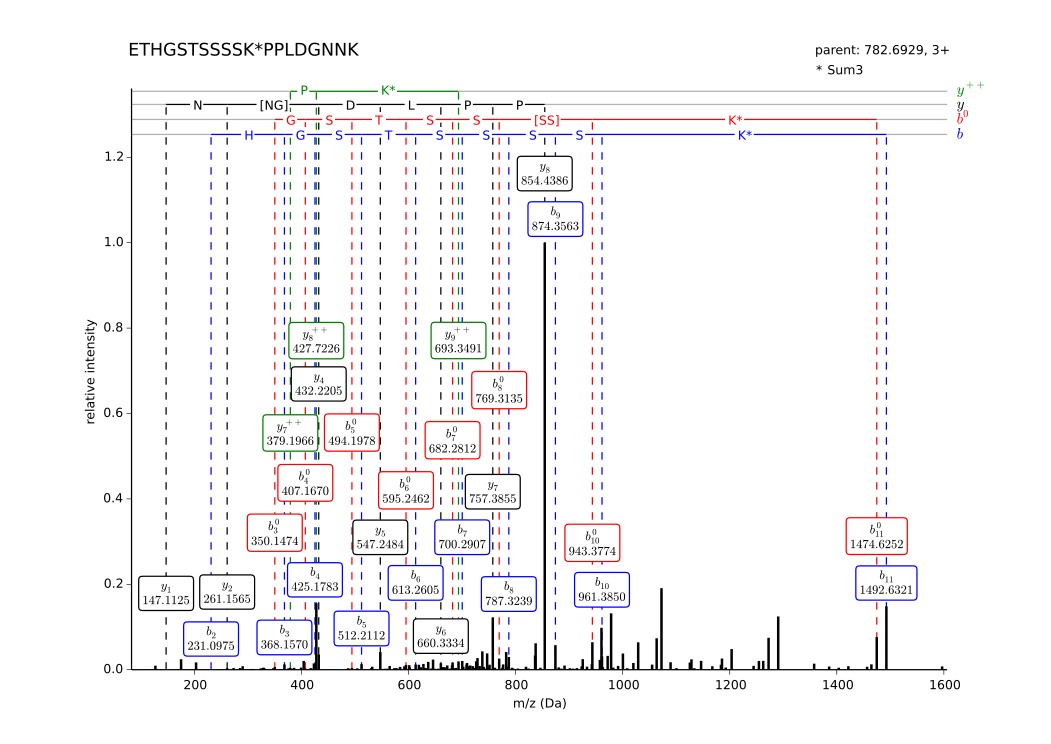

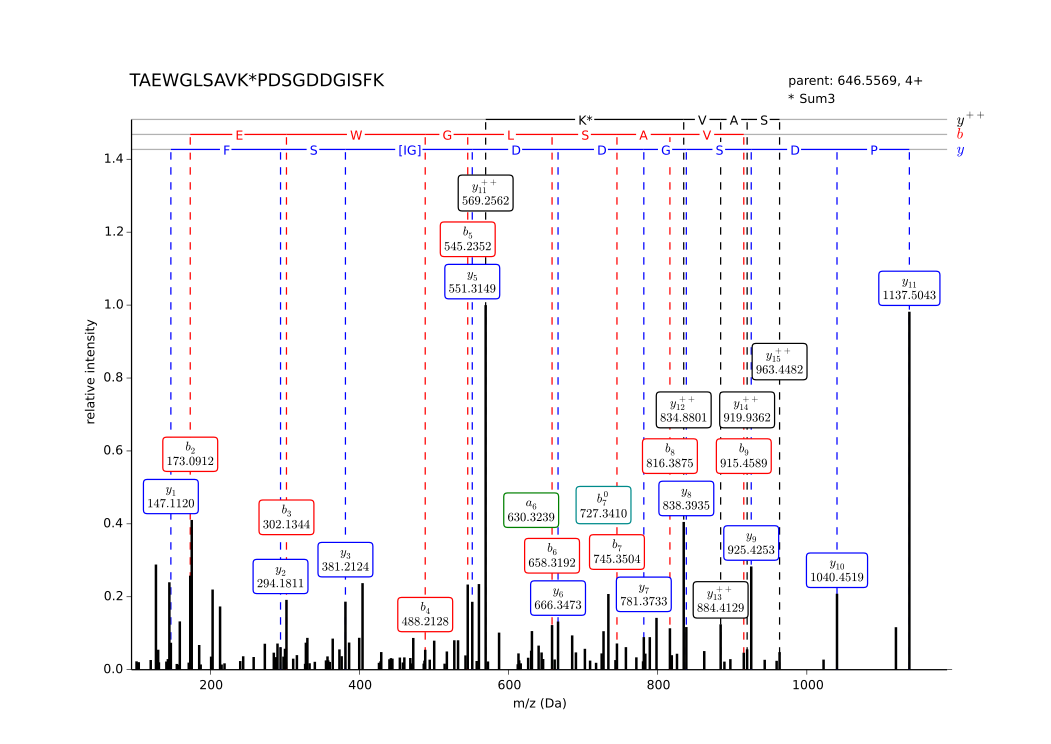

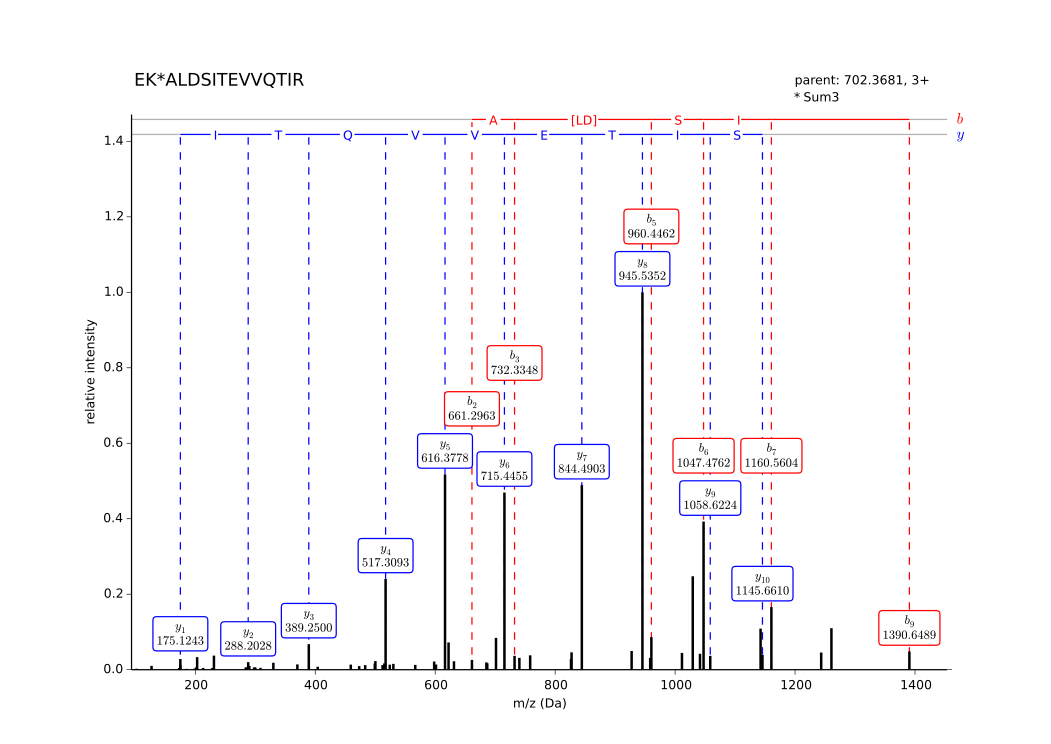

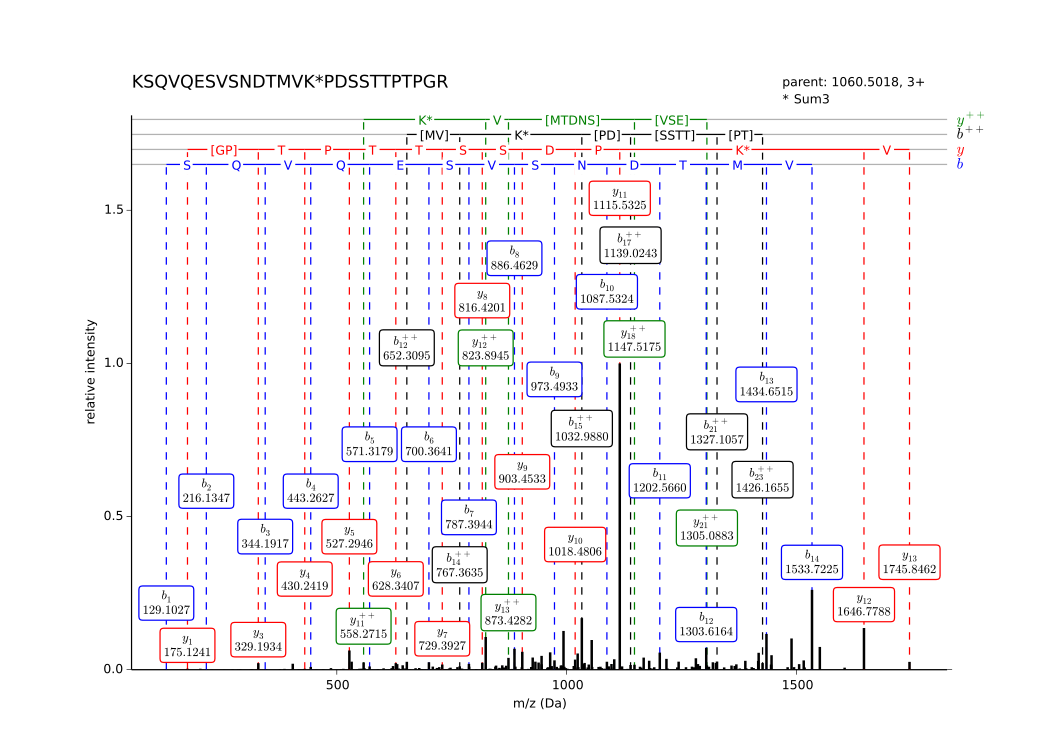

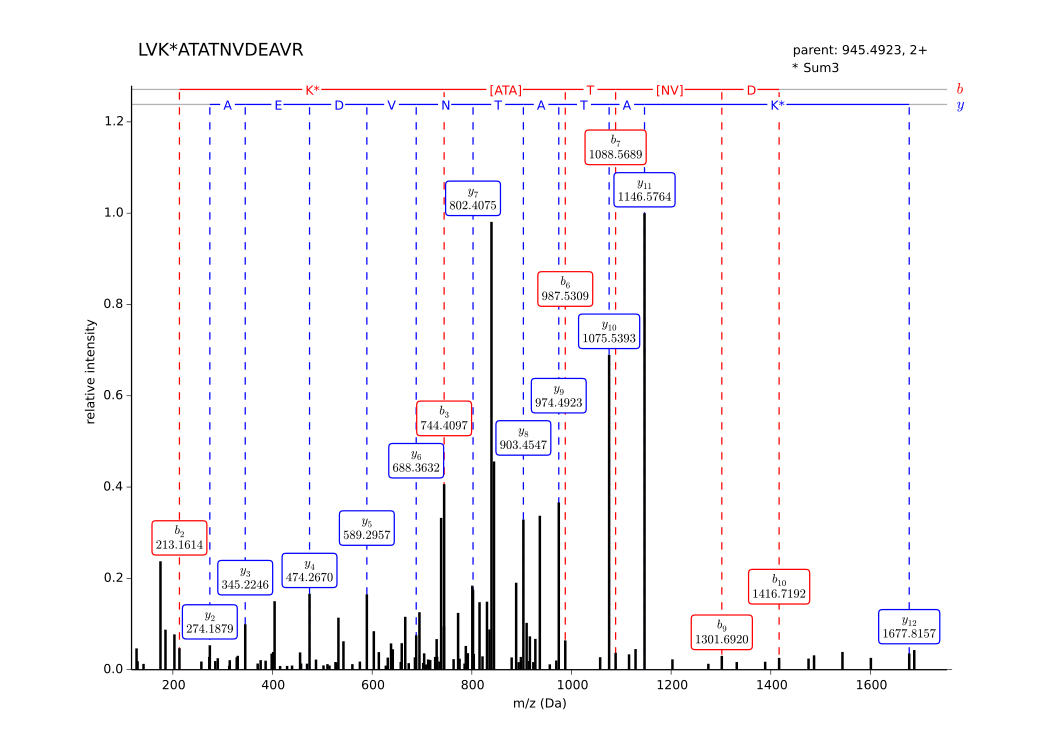

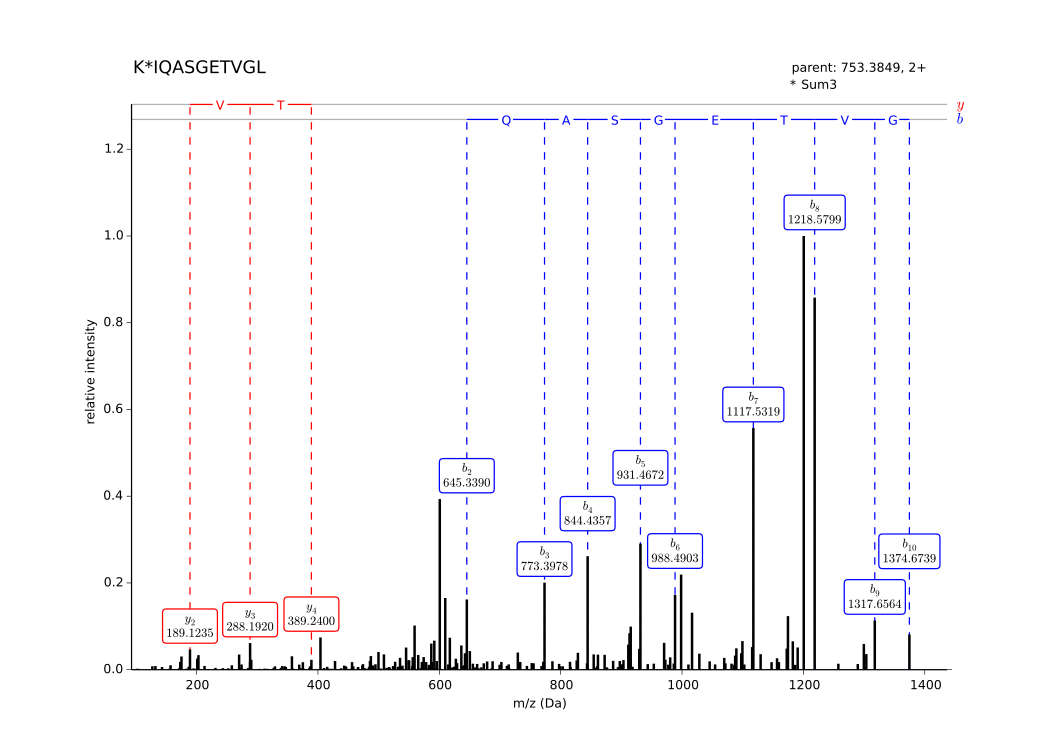


Fig. S13. Example MS spectra of tryptic digests of phot2 for sumoylation site mapping. SUMO3 modified phot2 peptides are linked via Lys with a fragment of SUMO3 – AMSGG.


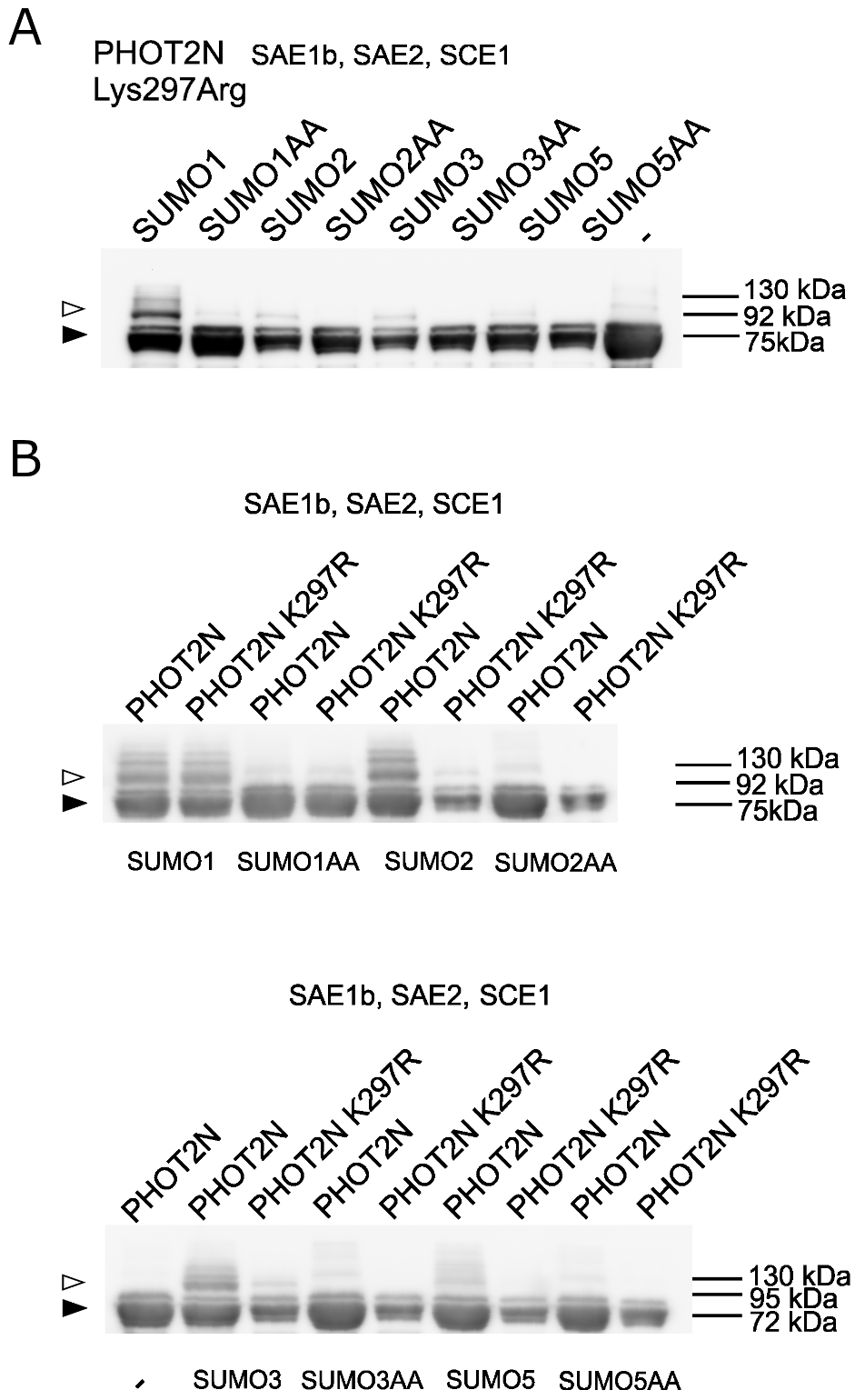


Fig. S14. The sumoylation pattern of the N-terminal PHOT2 part bearing the Lys297Arg mutation for different SUMO variants (A). Comparison between the native and mutated form of N-PHOT2 is shown in (B). The *Arabidopsis* SUMO modification system was reconstituted *in bacteria* by simultaneous overexpression of E1 (AtSAE1b, AtSAE2), E2 (AtSCE1a), and either AtSUMO1, AtSUMO2, AtSUMO3 or AtSUMO5 (native GG or mutated AA variants). PHOT2 fragments were purified using NiNTA and analyzed by Western blotting, with an anti-c-Myc antibody. In the negative control, only the N-terminal part of PHOT2 was overexpressed in *E. coli*. Arrowheads mark positions of non-sumoylated (black) or sumoylated (white) phototropin fragments.


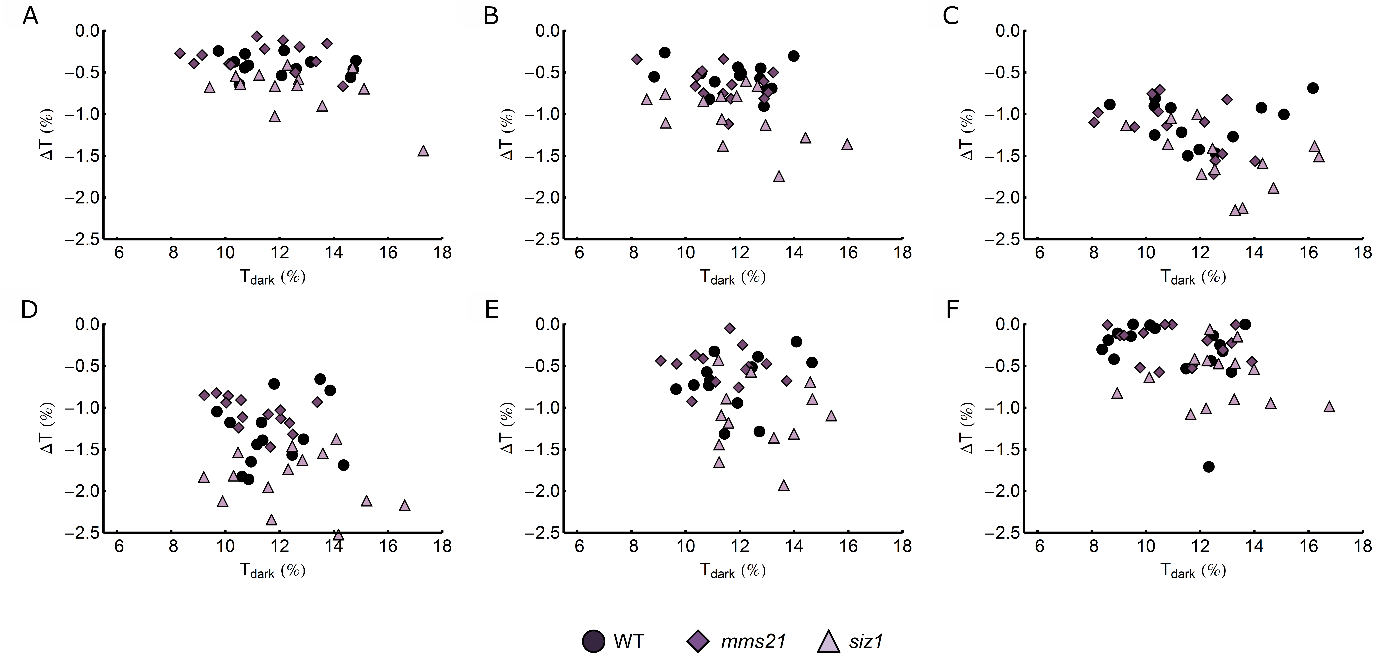


Fig. S15. The relation between the amplitude of transient accumulation and the dark transmittance levels in wild-type *Arabidopsis* and *mms21* and *siz1* mutants. Accumulation was triggered by a blue light pulse of 120 µmol m^−2^ s^−1^, whose duration was A: 0.1, B: 0.2, C: 1, D: 2, E:10 or F: 20 s.


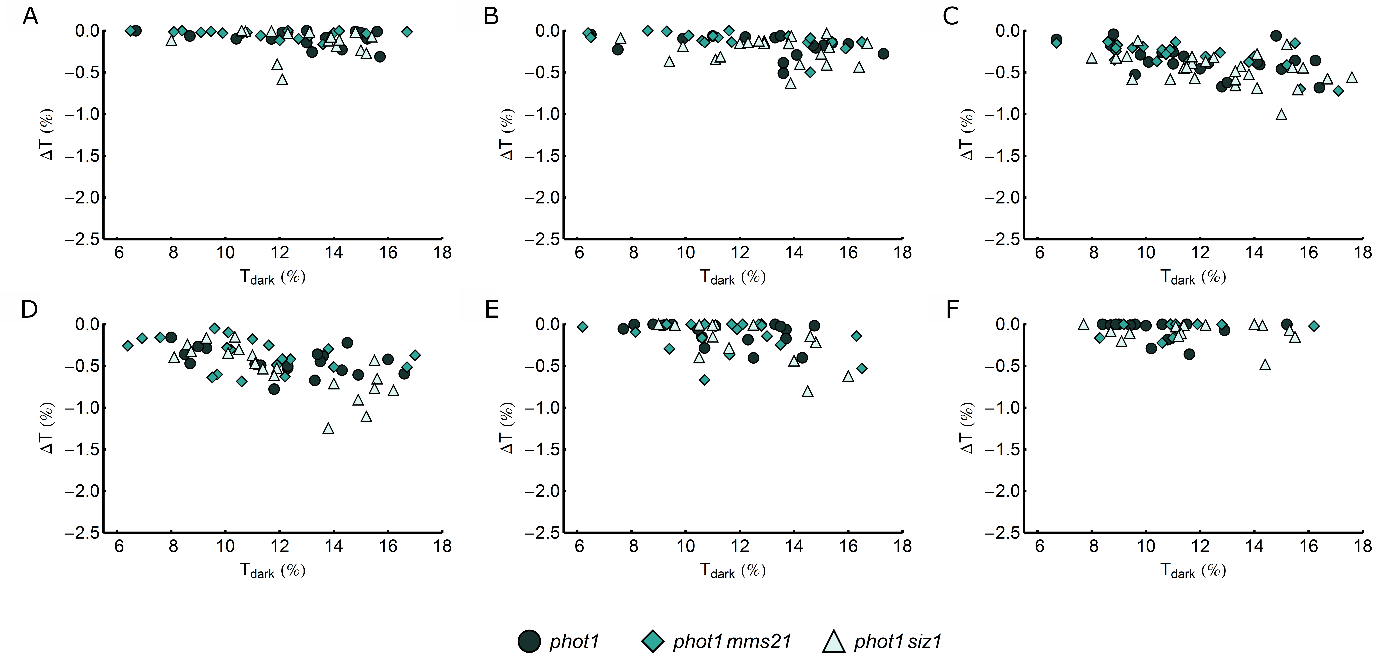


Fig. S16. The relation between the amplitude of transient accumulation and the dark transmittance levels in *Arabidopsis phot1,* *phot1mms21* and *phot1siz1* mutants. Accumulation was triggered by a blue light pulse of 120 µmol m^−2^ s^−1^, whose duration was A: 0.1, B: 0.2, C: 1, D: 2, E:10 or F: 20 s.


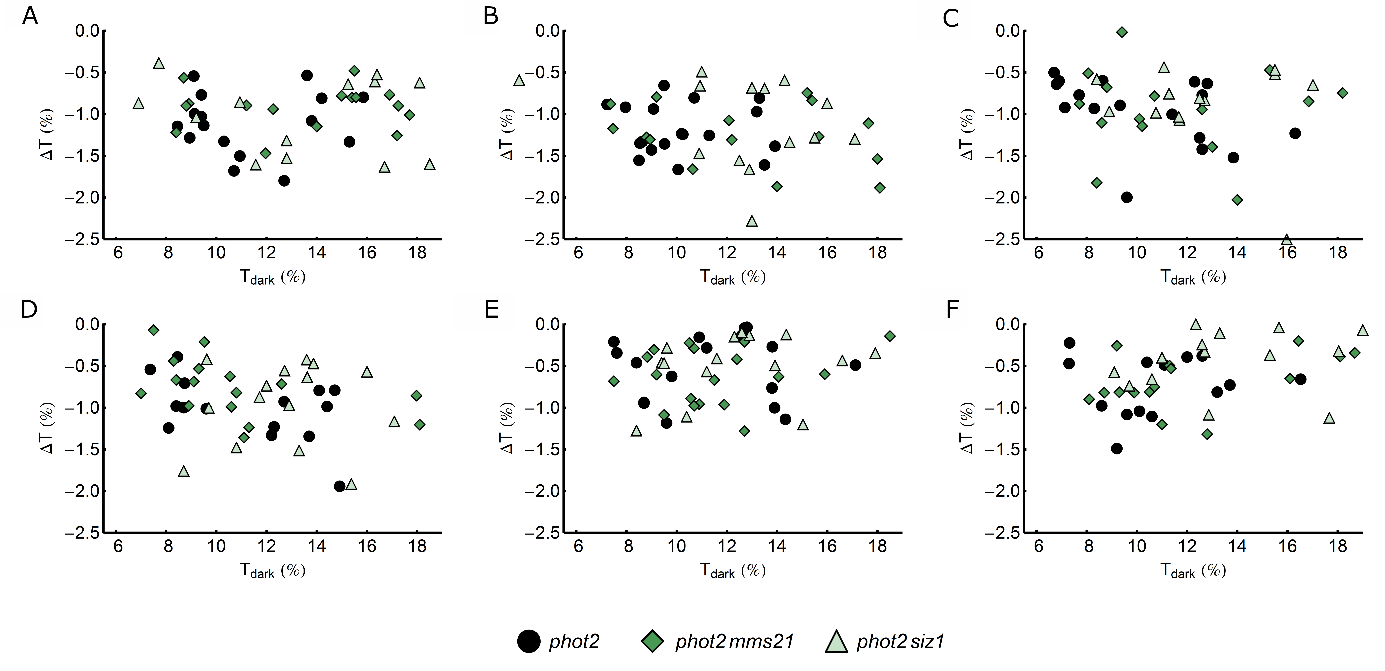


Fig. S17. The relation between the amplitude of transient accumulation and the dark transmittance levels in *Arabidopsis phot2,* *phot2mms21* and *phot2siz1* mutants. Accumulation was triggered by a blue light pulse of 120 µmol m^−2^ s^−1^, whose duration was A: 0.1, B: 0.2, C: 1, D: 2, E:10 or F: 20 s.

Table S1. Phot1 and phot2 sumoylation sites, predicted by the GPS-SUMO tool. Modified lysin residues are in bold. High stringency threshold was applied.

| Position of lysine | Sequence | P-value |
| --- | --- | --- |
| **phototropin1** | | |
| 125 | AEWGLVL**K**TDTKTGK | 0.033 |
| 284 | LLTIAPI**K**DESGKVL | 0.003 |
| 790 | GIIYRDL**K**PENVLIQ | 0.008 |
| **phototropin2** | | |
| 79 | EWGLSAV**K**PDSGDDG | 0.028 |
| 220 | LLTVTPI**K**DDQGNTI | 0.022 |
| 297 | VSNDTMV**K**PDSSTTP | 0.026 |
| 704 | GIVYRDL**K**PENILLK | 0.004 |
| 711 | KPENILL**K**KDGHIVL | 0.029 |
| 897 | DPNAKDI**K**WEDDGVL | 0.002 |

Table S2. Phot1 and phot2 putative sumoylation sites, predicted by the JASSA tool. Modified lysin residues are in bold. High score sites are shown.

| Position of lysine | Sequence | Type |
| --- | --- | --- |
| **phototropin1** | | |
| 284 | FWNLLTIAPI**K**DESGKVLKFI | Synergy control |
| 454 | RDERPESVDD**K**VRQKEMRKGI | Strong consensus inverted |
| **phototropin2** | | |
| 79 | RTAEWGLSAV**K**PDSGDDGISF | Negatively charged amino acid-dependent SUMOylation motif |
| 297 | QESVSNDTMV**K**PDSSTTPTPG | Extended phosphorylation-dependent SUMOylation motif |
| 897 | IEKDPNAKDI**K**WEDDGVLVNS | Strong consensus |

Table S3. Primer sequences used to test homozygosity of *Arabidopsis* plant lines.

| SUM1LP | TTTCGTGTAGCTGCGATTAGG |
| --- | --- |
| SUM1RP | TTATCTTTGCTCGCCATTAGC |
| SUM2LP | GTCGGAGAATCGGATTTCTTC |
| SUM2RP | TGAGGGTGTGTATTGGTGGAG |
| SUM3LP | CGATCTGCGATATAAGCGAAG |
| SUM3RP | AAAGCGCTCCACTTAAAAAGC |
| SUM5LP | AGCCTAATCTGTTTATCCCGC |
| SUM5RP | TTCGAATCATCCAATCTCCAG |
| SIZ1LP | TCCCTCGTAGACATCTGATGG |
| SIZ1RP | AAAGAGAGAGTGAGCGAAGGG |
| MMS21LP | AGTTCGCAGGTACAATGGATG |
| MMS21RP | CTAAATGCAGAGAATGCGGAC |
| PHOT1LP | GAGTTTGTACAAAAAAGCAGGC |
| PHOT1RP | TGGGTCAAAAAACATTTGTTTGCAG |
| PHOT2LP | GACGCTACACAGCCTCACTGTCCC |
| PHOT2RP | TCCCAACTGTCCCTCTGCCCTATT |
| LB1SAIL | GCCTTTTCAGAAATGGATAAATAGCCTTGCTTCC |

Table S4. Primer sequences used for Gateway cloning.

| siz1_F | GGGGACAAGTTTGTACAAAAAAGCAGGCTCGATGGATTTGGAAGCTAATTGTAAGG |
| --- | --- |
| siz1_R | GGGGACCACTTTGTACAAGAAAGCTGGGTCCTCAGAATCCGAGTCAATGGAG |
| mms21_F | GGGGACAAGTTTGTACAAAAAAGCAGGCTCGATGGCGTCGGCGTCCTCGTC |
| mms21_R | GGGGACCACTTTGTACAAGAAAGCTGGGTCATCTTCATCCACATCTTCTG |
| sumo1_F | GGGGACAAGTTTGTACAAAAAAGCAGGCTCCATGTCTGCAAACCAGGAGGAAG |
| sumo1_R | GGGGACCACTTTGTACAAGAAAGCTGGGTCTTAGCCACCAGTCTGATGGAGCATC |
| sumo1AA_R | GGGGACCACTTTGTACAAGAAAGCTGGGTCTTAAGCGGCAGTCTGATGGAGCATC |
| sumo2_F | GGGGACAAGTTTGTACAAAAAAGCAGGCTCCATGTCTGCTACTCCGGAAGAAG |
| sumo2_R | GGGGACCACTTTGTACAAGAAAGCTGGGTCTTAACCACCAGTCTGATGAAGCAT |
| sumo2AA_R | GGGGACCACTTTGTACAAGAAAGCTGGGTCTTAAGCAGCACCACCAGTCTGATGAAG |
| sumo3_F | GGGGACAAGTTTGTACAAAAAAGCAGGCTCCATGTCTAACCCTCAAGATGAC |
| sumo3_R | GGGGACCACTTTGTACAAGAAAGCTGGGTCTTAACCACCACTCATCGCCCGGCAC |
| sumo3AA_R | GGGGACCACTTTGTACAAGAAAGCTGGGTCTTAAGCAGCACTCATCGCCCGGCAC |
| sumo5_F | GGGGACAAGTTTGTACAAAAAAGCAGGCTCCATGGTGAGTTCCACAGACAC |
| sumo5_R | GGGGACCACTTTGTACAAGAAAGCTGGGTCTTAGCCACCACCAAGTTCCATGACC |
| Sumo5AA_R | GGGGACCACTTTGTACAAGAAAGCTGGGTC TTAAGCAGCACCAAGTTCCATG |

Table S5. Plasmids used for preparation of BiFC constructs.

| Plasmid name | Short description | Reference |
| --- | --- | --- |
| pDONR221 | Gateway entry vector |  |
| pH7m34GW | MultiSite Gateway intermediary vector (three fragment recombination plus terminator) backbone vector for C-terminal fusion, used to obtain 35S-protein-nGFP | (Karimi *et al.*, 2005) |
| pK7m34GW | MultiSite Gateway intermediary vector (three fragment recombination plus terminator), backbone vector for C-terminal fusion used to obtain 35S-protein-cGFP | (Karimi *et al.*, 2005) |
| pEN-L4-2-R1 | Gateway entry vector with 35S promoter used for multisite gateway reaction | (Karimi *et al.*, 2007) |
| pEN-R2-teGFP-L3 | Gateway entry vector with cGFP used for multisite gateway reaction to obtain cGFP fusion at the C-terminus of the protein | (Karimi *et al.*, 2007) |
| pEN-L4-heGFP-R1 | Gateway entry vector with nGFP used for multisite gateway reaction to obtain nGFP fusion at the N-terminus of the protein | (Karimi *et al.*, 2007) |
