# Supplementary material for: Phototropin Interactions with SUMO Proteins
Source: Plant Cell Physiol. 2021 Feb 17;62(4):693–707. doi: 10.1093/pcp/pcab027 (PMC8462379; doi:10.1093/pcp/pcab027)
Supplement: pcab027_Supp [file pcab027_supp.zip › pcp-2020-e-00456-File013.docx]

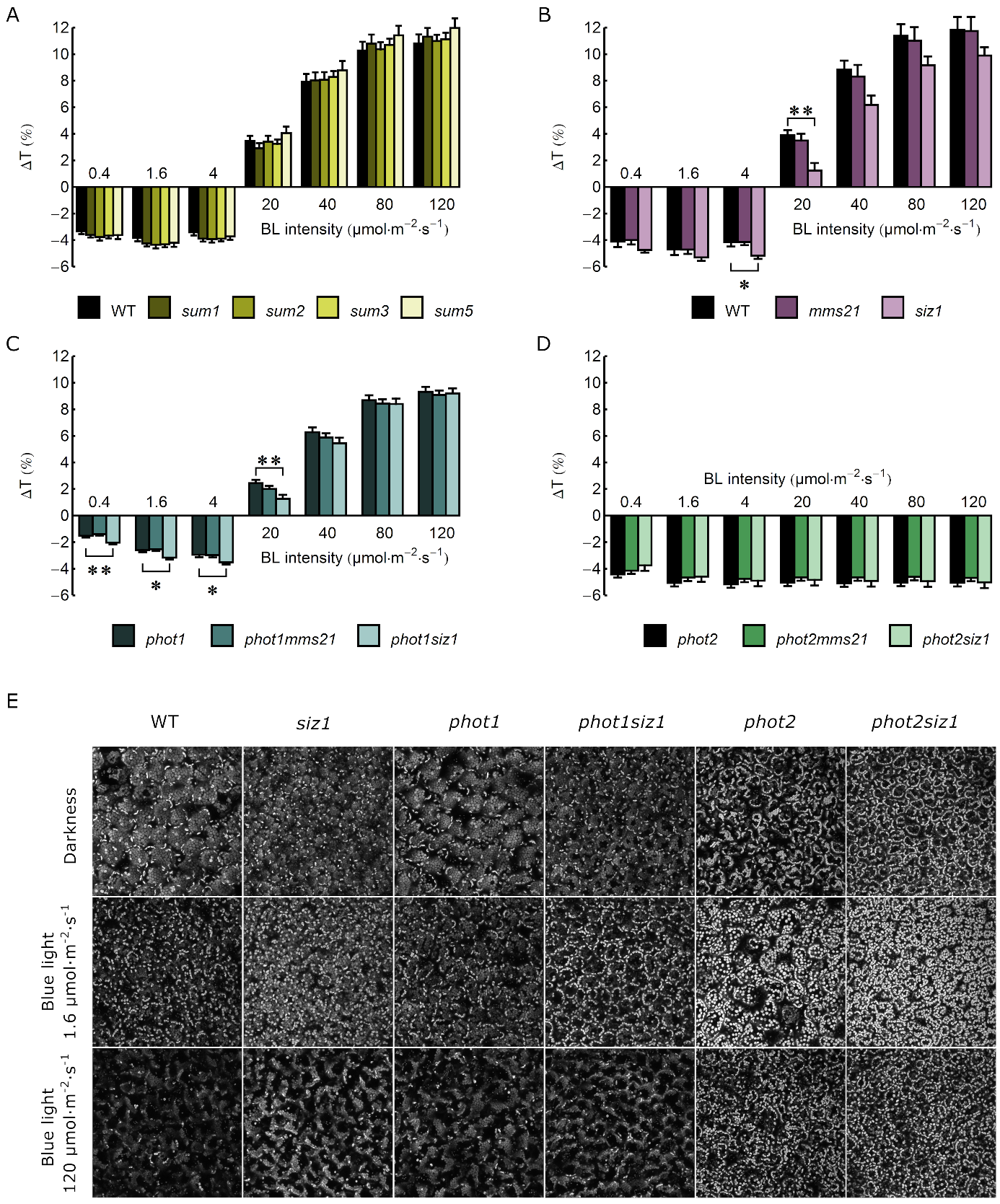


Fig. 1. A,B,C,D: Amplitudes of transmittance changes ΔT due to chloroplast movements in leaves illuminated with continuous blue light of increasing intensity (0.4 – 120 µmol·m^-2^·s^-1^) in A: *sum* mutants, B: *siz1* and *mms21* ligase mutants, C: *phot1siz1* and *phot1mms21* mutants, D: *phot2siz1* and *phot2mms21* mutants. Asterisks indicate statistically significant differences between mutant lines and the control (*phot1* for *phot1mms21* and *phot1siz1*, *phot2* for *phot2mms21* and *phot2siz1*, the wild type for other lines), as tested with the Dunnett’s test (*P=0.01–0.05; **P=0.001–0.01, ***P<0.001). Error bars = SE. E: Blue light-induced chloroplast arrangements in palisade cells of *Arabidopsis* leaves of wild type, *siz1, phot1, phot2*, *phot1siz1*, and *phot2siz1* plants. Leaves were kept in darkness or irradiated with blue light (LED 460 nm, 1W, epiLED) of 1.6 or 120 µmol·m^−2^·s^−1^ for 50 min. Chloroplast arrangements were then examined with a confocal microscope, using chlorophyll autofluorescence (633 nm excitation, 661 – 721 nm emission). Maximum intensity projections were calculated from Z-stacks, which spanned whole depth of the epidermis and palisade parenchyma, starting from the leaf upper surface. The fluorescence from chloroplasts located at the bottom of palisade cells is less intense due to attenuation of excitation light.


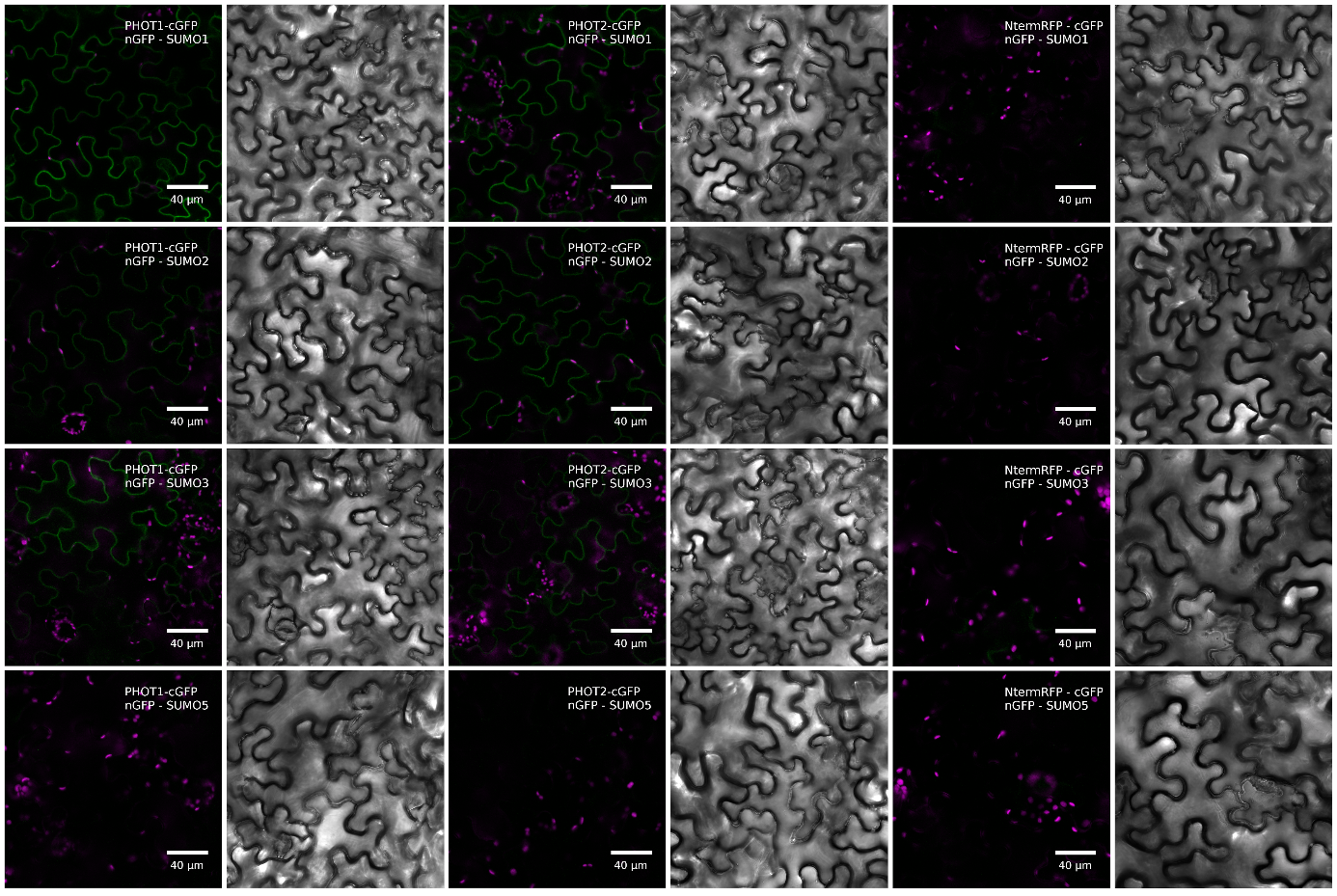


Fig. 4. Confocal microscopy images of N. benthamiana epidermal cells transiently co-expressing phototropins fused with the C-terminal green fluorescent protein fragment (cGFP) and SUMO isoforms fused with the N-terminal fragment of GFP (nGFP). Control plants expressed the first 150 amino acids from the N-terminal part of the red fluorescent protein fused with the C-terminal GFP fragment and the N-terminal GFP fragment fused with SUMO isoforms. Chlorophyll autofluorescence is in magenta and reconstituted GFP fluorescence is in green. Gray-scale images show transmitted light. The results represent one of three independent biological replicates.


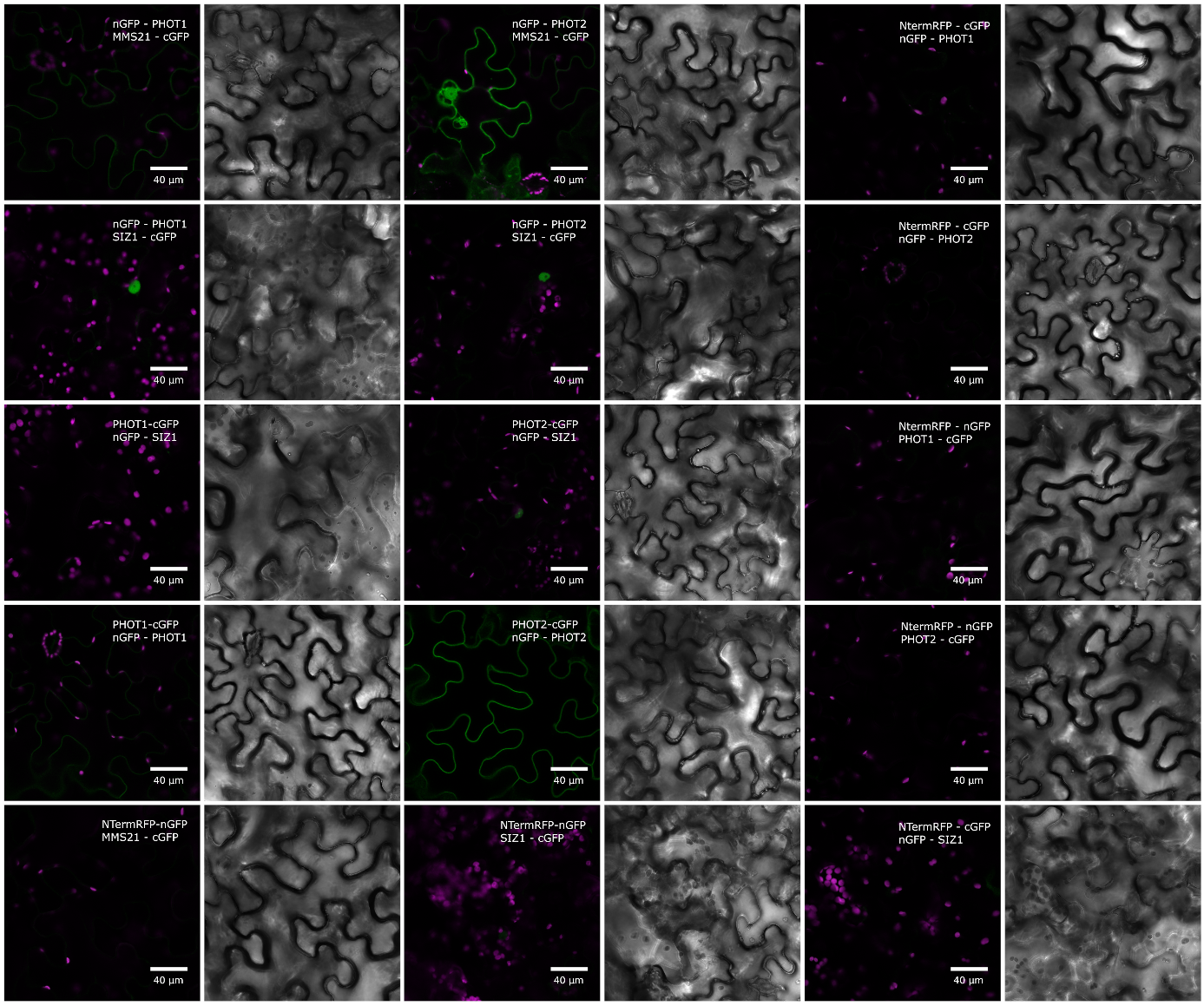


Fig. 5. Confocal microscopy images of *N. benthamiana* epidermal cells transiently co-expressing phototropins fused with the N- (nGFP) or C-terminal (cGFP) GFP fragment and E3 ligases fused with nGFP or cGFP. Negative control plants expressed the first 150 amino acids from the N-terminal part of the red fluorescent protein fused with the C-terminal or N-terminal GFP fragment and the tested protein partners fused with appropriate GFP fragments. Leaves co-expressing phototropins fused with nGFP and with cGFP were used for positive controls, as phototropins are known to form dimers. Chlorophyll autofluorescence is in magenta and reconstituted GFP fluorescence is in green. Gray-scale images show transmitted light. The results represent one of three independent biological replicates.
